# Exfoliated WS_2_-Nafion Composite based Electromechanical Actuators

**DOI:** 10.1038/s41598-017-14806-x

**Published:** 2017-11-03

**Authors:** Masoud S. Loeian, Dominika A. Ziolkowska, Farhad Khosravi, Jacek B. Jasinski, Balaji Panchapakesan

**Affiliations:** 1 Small Systems Laboratory Department of Mechanical Engineering, Worcester Polytechnic Institute, Worcester, MA 01609 USA; 2 Conn Center for Renewable Energy Research, University of Louisville, Louisville, KY 40292 USA; 3 Faculty of Physics, University of Warsaw Pasteura 5, 02-093 Warsaw, Poland

**Keywords:** Two-dimensional materials, Two-dimensional materials

## Abstract

The ability to convert electrical energy into mechanical motion is of significant interest in many energy conversion technologies. Here, we demonstrate the first liquid phase exfoliated WS_2_-Nafion nanocomposite based electro-mechanical actuators. Highly exfoliated layers of WS_2_ mixed with Nafion solution, solution cast and doped with Li^+^ was studied as electromechanical actuators. Resonant Raman spectroscopy, X-ray photo-electron-spectroscopy, differential scanning calorimetry, dynamic mechanical analysis, and AC impedance spectroscopy were used to study the structure, photoluminescence, water uptake, mechanical and electromechanical actuation properties of the exfoliated nanocomposites. A 114% increase in elastic modulus (dry condition), 160% increase in proton conductivity, 300% increase in water uptake, cyclic strain amplitudes of ~0.15% for 0.1 Hz excitation frequency, tip displacements greater than nanotube-Nafion and graphene-Nafion actuators and continuous operation for more than 5 hours is observed for TMD-Nafion actuators. The mechanism behind the increase in water uptake is a result of oxygen atoms occupying the vacancies in the hydrophilic exfoliated flakes and subsequently bonding with water, not possible in Nafion composites based on carbon nanotube and graphene.

## Introduction

The ability to convert electrical energy into mechanical motion is highly desirable for energy conversion, actuation, robotics and reconfigurable technologies. Today, a wide variety of smart technologies have been developed based on the conversion of electrical to mechanical energy which is used broadly from simple home blenders to complex aerospace technologies. Materials such as piezoelectrics^1^, ferroelectrics^2^, shape memory alloys^3^ and electroactive polymers^4–7^ are used as actuators that convert electrical energy into mechanical output. Many applications of such materials have been proposed including atomic force microscopes^8^, biomimetic robots^9^, and artificial muscles^6^. With the advent of nanomaterials such as carbon nanotubes^10^ and graphene^11^, they have also been explored in their native form as electrical actuators^12^ and also by mixing them in different matrices to make nanocomposites^13–15^.

Here, we describe the working of a new type of actuator based on Nafion and Transition Metal Dichalcogenides (TMDs) based nanocomposites. Nafion is a sulfonated tetrafluoroethylene based fluoropolymer-copolymer with high porosity and unique ionic properties and was initially developed as electrolytic separators^16^. They have found applications in diverse areas such as ion exchange membranes^17^, proton conductors^18^, drug delivery^19^, batteries^20^ and in fuel cells^21^ due to their excellent mechanical properties, stability, electro-activity, water nanochannels and low cost. In the past, Nafion was mixed with carbon nanotubes^14^, graphene^15^, and metallic nanoparticles^22^ to improve actuation and bending characteristics of electro-mechanical actuators. Recently, semiconductors such as TMDs have found various applications as transistors^23^, optoelectronic devices^24^, photo-thermal actuators^25^ and solar cells^26^. However, they have not been evaluated as electro-active materials in Nafion polymer actuators.

Transition Metal Dichalcogenides (TMDs) are the class of 2D layered materials with transition metal layer between two chalcogen layers. The atoms within the layers are bonded through strong covalent bonds^27^. The weak van der Waals bonding between the layers enables the triple layers to be mechanically/chemically shear exfoliated. The in-plane structure of WS_2_ is determined by strong covalent bonds resulting from the overlap between the *4d* and *3p* electron orbitals of W and S respectively^28^. The elastic modulus and the breaking strength of an ideal defect-free single-layer WS_2_ are similar to those of MoS_2_ and is expected to reach the theoretical limits of E^2D^/9, where E^2D^ is the in-plane stiffness of the 2D layered material^29,30^. The ultra-high strength originates from the *p* orbitals of the chalcogen atoms which give rise to the σ bonds^31^. The large strength, ability to modulate the conduction of electrons through doping and enhanced water uptake in TMDs can result in their application for Nafion based electro-mechanical actuation technologies.

Layered materials represent a diverse and largely untapped source of two-dimensional (2D) systems with exotic electronic properties and high specific surface areas that are important for sensing^32^, catalysis^33^, energy storage^34^, and actuation applications^35^. Similar to graphene, the bright future of 2D TMDs can only be realized through large-scale exfoliation^36^. Making single/bi/few layers TMDs has been attempted using mechanical exfoliation^37^, chemical synthesis^38^, and liquid phase exfoliation^36^. However, Nafion-TMD composites as proposed here and smart materials and systems based on them cannot be achieved using scotch tape exfoliation methods. Liquid-phase exfoliation is the strategy that is used for large volume processing for applications such as composites, batteries, and thin films to name a few^36^. Initial work by Coleman *et al*. has shown the liquid phase exfoliation of few layers using a wide variety of solvents. Typically, high yield of few layers and low yield of single layers are obtained^36^.

By utilizing liquid phase exfoliation, the solution casting of nanocomposite fabrication and electromechanical testing, we present the structural properties, interaction mechanisms and actuation properties of WS_2_-Nafion nanocomposites. The advantages of our fabrication methods are as follows: (1) the liquid phase exfoliation resulting in high quality single to few layers with distinctive direct electron transition peaks and bandgaps; (2) phase desegregated nanocomposites due to excellent dispersion of exfoliated additives and improvement in mechanical properties, (3) access to the unique layer dependent electronic/optical properties of WS_2_ layers inside the nanocomposite (ex: photoluminescence), (4) the design enables scalable and flexible process for developing stimuli-responsive wearable and energy harvesting devices in water and air, (5) the exfoliated WS_2_ nanosheets enhanced water retention properties of Nafion by 300% that served to enhance and tune actuation behavior of Nafion composites.

## Results and Discussion

**Figure 1:**
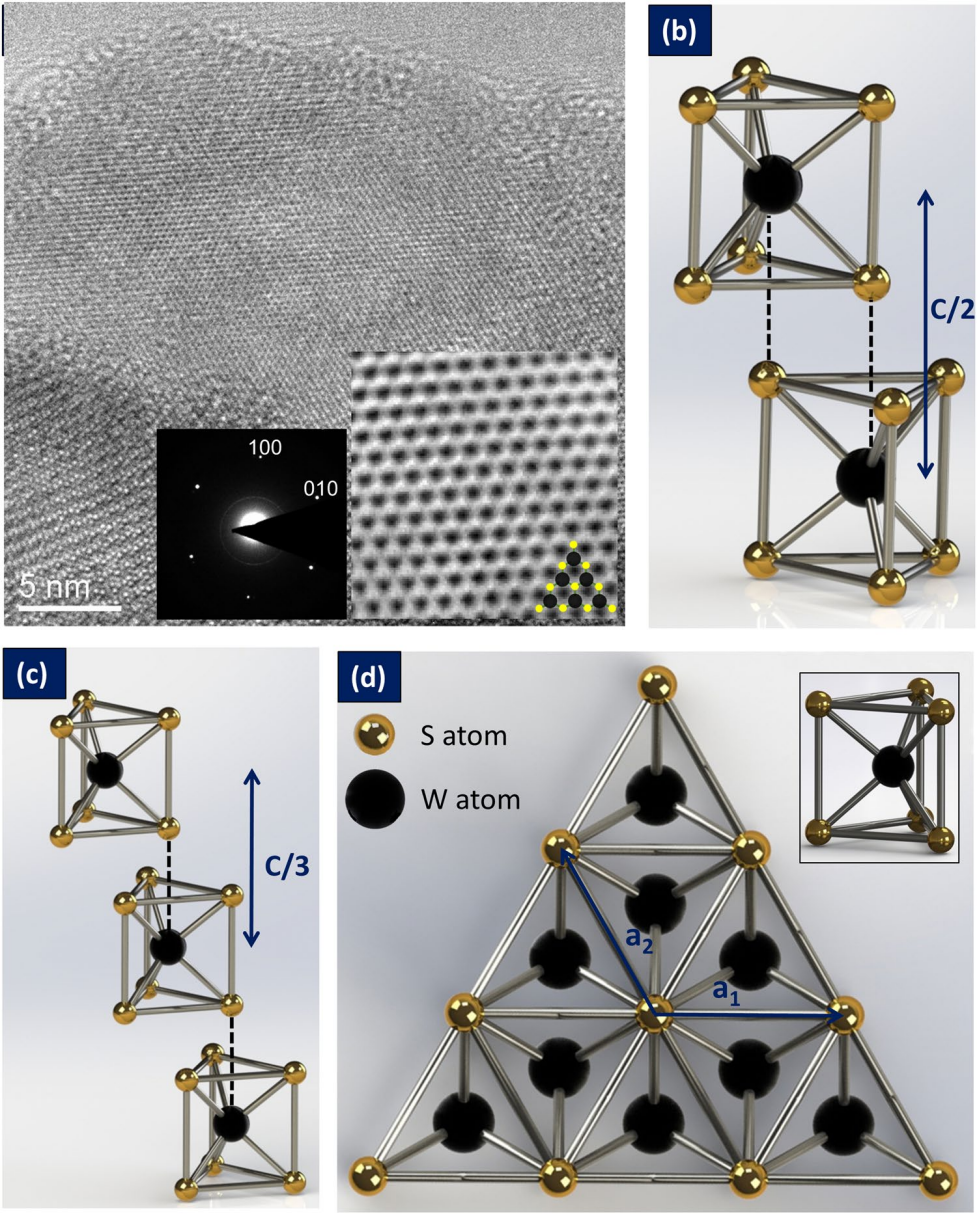
Structure of WS_2_: (**a**) High-resolution TEM image of the WS_2_ single layer; insets are the SAED pattern and atomic structure of monolayer WS_2_ with atoms indicated in black and yellow; (**b**) Schematic of the semiconducting phase of WS_2_ for 2H-WS_2_; (**c**) 3R-WS_2_; (**d**) 2D structure of WS_2_ monolayer seen from above in triangular configuration.

Figure 1 presents the High-Resolution Transmission Electron Micrograph (HRTEM) of the 2H-WS_2_ single layer with Selected Area Diffraction Pattern (SAED). WS_2_ belongs to the same family of TMDs as MoS_2_ and can be mechanically exfoliated using scotch tape and liquid phase exfoliation techniques. In the WS_2_ lattice, each W atom is located at the center of a trigonal prism created by six S atoms. The lattice  parameters of WS_2_ was reported to be: a = b = 3.153 Å and c = 12.3 Å^39^. A single layer of WS_2_ is ~0.7 nm thick^40^ and can be exfoliated using scotch tape and liquid phase exfoliation techniques^36^. The HRTEM was conducted to ascertain the structure of the WS_2_ presented in Fig. 1 with atoms of W and S atoms indicated in the monolayer inset. The Selected Area Electron Diffraction (SAED) pattern is presented as inset in Fig. 1(a) with (100) and (010) diffraction spots marked. The d-spacing of (100)-type planes were measured from both HRTEM and SAED images as d = 2.76 ± 0.04 Å and d = 2.77 ± 0.06 Å respectively. Within experimental errors, this value agree with the d = 2.73 Å value obtained from the lattice parameter a = 3.1532 Å, reported in literature for 2H-WS_2_^39^. The distances a_1_, a_2_ and a_3_ measured from this image are close to 3.15 Å value of the W–W interatomic distances in WS_2_^41^. The bulk form of WS_2_ has an indirect band gap of ~1–1.4 eV^27^, and the features of the WS_2_ band structure are similar to those of MoS_2_ where a direct and indirect gap coexist irrespective of thickness^41^. A direct gap exists at the K point of the Brillouin zone between the spin–orbit split valence band and the doubly degenerate conduction band^41^. On the other hand, indirect gap forms between a local conduction band minimum at a midpoint between Γ and Κ and the valence band maximum at the Γ point^41^. As the number of layers decreases in WS_2_, the material changes into a direct band gap semiconductor with E_g_~1.9–2.0 eV exhibiting photoluminescence. The elastic modulus of chemical vapor deposited WS_2_ has been reported to be ~170 N/m similar to 2H-MoS_2_, and about half the value of graphene which is the strongest 2D material^42^. While materials such as MoS_2_ have been intensely researched in recent years, there are only a few reports on structure, properties, and especially applications of WS_2_ and other layered 2D crystals^41^. The high strength, electronic properties, and excellent water uptake properties call for the development of ionic electro-mechanical actuators based on WS_2_.

**Figure 2:**
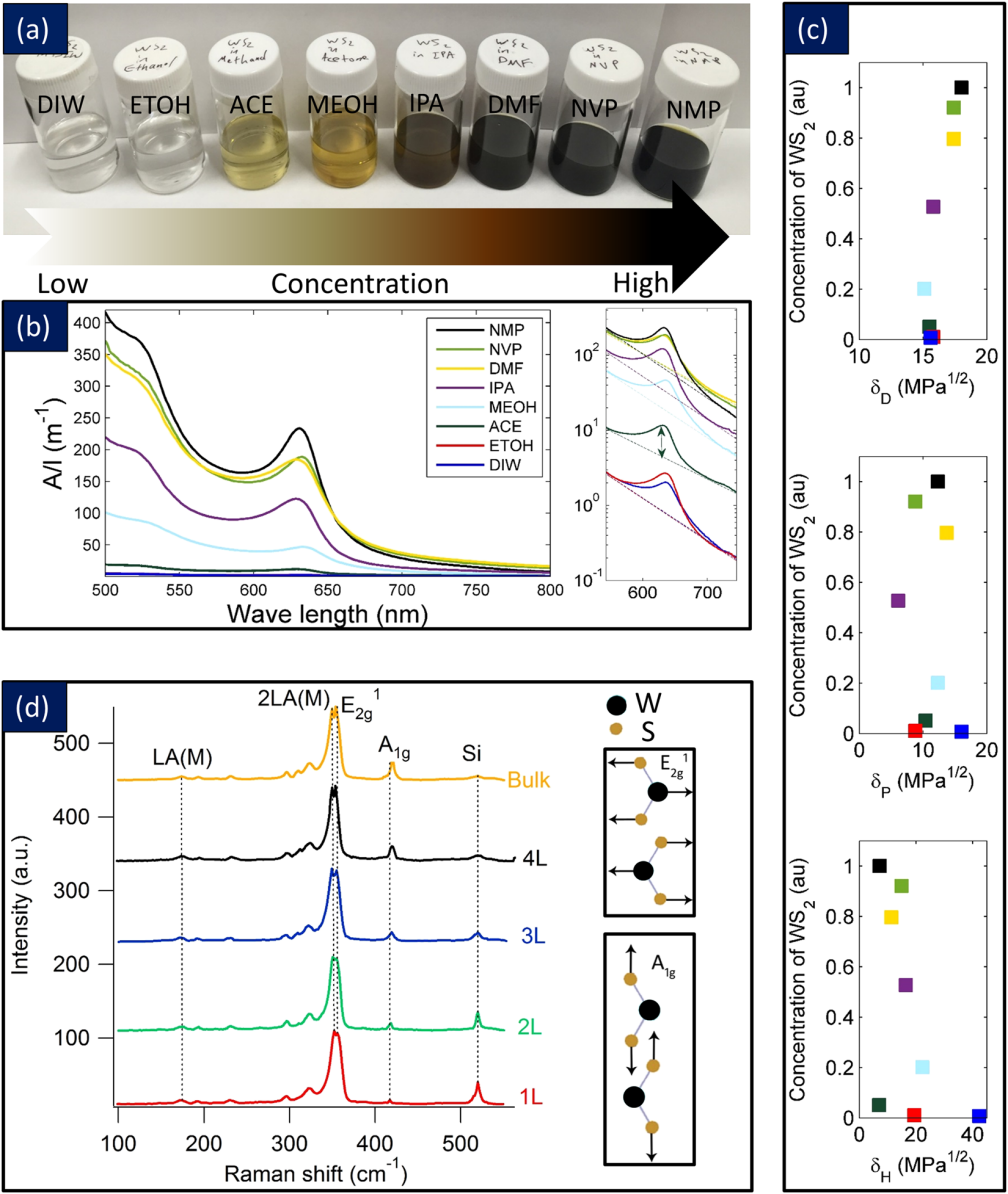
Liquid Phase Exfoliation: (**a**) Photograph of WS_2_ dispersions after exfoliation and centrifugation in different solvents; (**b**) UV- Visible spectra of the WS_2_ dispersions including their scattering components, (**c**) The correlation between the exfoliated solutions concentration and Hansen parameters for solvents which were employed, (**d**) Raman spectra (λ_exc_ = 532 nm) of WS_2_ few layers deposited on silicon wafer suggesting bulk, 4 L, 3 L, 2 L and 1 L flake.

The starting point for our electro-mechanical actuators is the liquid phase exfoliation of WS_2_^36^ powders in a variety of solvents followed by centrifugation and separation of the layers as presented in Fig. 2(a). Eight different solvents were used: deionized water (DIW), ethanol (EtOH), acetone (Ace), methanol (MeOH), isopropyl alcohol (IPA), dimethyl formamide (DMF), N-vinylpyrrolidone (NVP) and N-Methyl Pyrrolidone (NMP). These enabled varying levels of exfoliation of the layers as presented n Fig. 2(a). Solvents such as NMP, NVP, and DMF gave rise to a high degree of exfoliation, and the color of the solutions suggest a high density of exfoliated layers. Figure 2(b) presents the UV-Visible spectroscopy of the exfoliated layers in different solvents. Lambert-Beer’s law as in *A*/*l = *α*C*, where *A*/*l* is the absorbance per unit length, α is the extinction coefficient, and *C* is the concentration was used to calculate extinction coefficients. The *A*/*l* scaled linearly with *C* provided the α values for the different types of solution. Table 1 presents the calculated extinction coefficients for different solvents. The extinction coefficient increase with a decrease in the number of layers in different solvents. Figure S1 (Suppl.) provides the optical absorption without the scattering component. The two peaks marked A (1.9 eV), and B (2.1 eV) correspond to the direct exciton transition at the K point (peaks assigned to excitons involving the conduction band and the two valence bands split due to spin-orbit coupling)^43^.

**Table 1:** Absorption cut off and peak wavelength for different solvents.

Solvent	Cut off wave length (nm)	Cut off bandgap (eV)	Peak wave length (nm)	Peak bandgap (eV)	Concentration (µg/ml)	Extinction Coefficient (α)
DIW	787	1.576	634.0	1.956	1.17	812
ETOH	771	1.608	633.6	1.957	1.71	861
ACE	747	1.660	628.8	1.972	11.22	1123
MEOH	797	1.556	632.5	1.960	32.58	1036
IPA	791	1.568	628.6	1.973	78.73	1245
DMF	788	1.574	629.2	1.971	100.69	1312
NVP	796	1.558	632.1	1.962	108.93	1276
NMP	771	1.608	631.0	1.965	139.00	1346

Detailed analysis, within the framework of Hansen solubility parameter theory, shows successful solvents to be those with dispersive, polar, and H-bonding components of the cohesive energy density within certain well-defined ranges^44^. The dispersion of nanomaterials in liquids is partially predicted by the theory of Hansen solubility parameters (HSP), which is a semi-empirical correlation developed to explain dissolution behavior. Three HSP parameters are used to describe the character of a solvent or material: δ_D_, δ_P_, and δ_H_, which are the dispersive, polar, and hydrogen bonding solubility parameters, respectively. Each solubility parameter represents the square root of the contribution to the cohesive energy density, and the sum of their squares equals the square of the Hildebrand solubility parameter: δ_T_^2^ = δ_D_^2^ + δ_P_^2^ + δ_H_^2^. Typical values for graphene analogs are δ_D_ (16–19 MPa^1/2^), δ_P_ (4–12 MPa^1/2^), and δ_H_ (2–19 MPa^1/2^)^44^. These were plotted for all the solvents suggesting an excellent agreement between theory and experiments. Figure 2(c) shows the dispersed concentration of different solvents versus Hansen solubility parameters. Based on these results, solvents that have H-parameter (δ_H_) around 8, P-parameter (δ_P_) around 13, and D-parameter (δ_D_) around 18 are excellent choices as a solvent for exfoliation of WS_2_ as seen in our results and others^36^.

Figure 2(d) presents the Raman spectroscopy of the WS_2_ layers, and here we compared it to bulk. The spectra include the first order modes at the Brillouin zone center E_2g_ and A_1g_ plus a zone-edge mode activated by the disorder. This is identified as the longitudinal acoustic mode at the M point, LA(M). The longitudinal acoustic phonons LA (M) are the in-plane collective oscillations of the atoms in the lattice, similar to the sound waves. They are periodic compressions and expansions of the lattice that occur along the direction of propagation. For λ_exc_ = 532 nm, it is striking to see the LA(M) at 176 cm^−1^ and the first order modes E^1^_2g_ at 350 cm^−1^ and A_1g_ mode at 417 cm^−1^ for the WS_2_ single layer. It should be noted that for λ_exc_ = 532 nm, the 2LA (M) mode is very close to the first-order E^1^_2g_(Γ) mode at 350 cm^−1^. There is a red shift in E^1^_2g_ mode and blue shift in the A_1g_ mode with decreasing number of layers that is the signature in the Raman spectra of WS_2_ few layers. The blue shift of the A_1g_ mode is consistent with the increasing restoring force caused by van der Waals interactions established between the layers, and it is in agreement with previous results reported for MoS_2_^45^ and WS_2_^46^. The change in E_2g_ with decreasing number of layers is quantified, and these results are presented in Table 2. The Raman shift is definitively indicative of 1 L, 2 L, 3 L, 4 L, and bulk. These are in excellent agreement with the other published papers on Raman spectroscopy of WS_2_ and MoS_2_ which are similar in structure^45–47^.

**Table 2:** The Raman shift for major picks in Raman spectra of WS_2_ flakes.

Raman mode	E2g1 Raman shift (cm^−1^)	A1g Raman shift (cm^−1^)
Single layer	355.76	417.20
Bilayer	354.75	418.29
3 layers	354.70	419.17
4 Layers	354.57	420.35
Bulk	353.62	420.42

**Figure 3:**
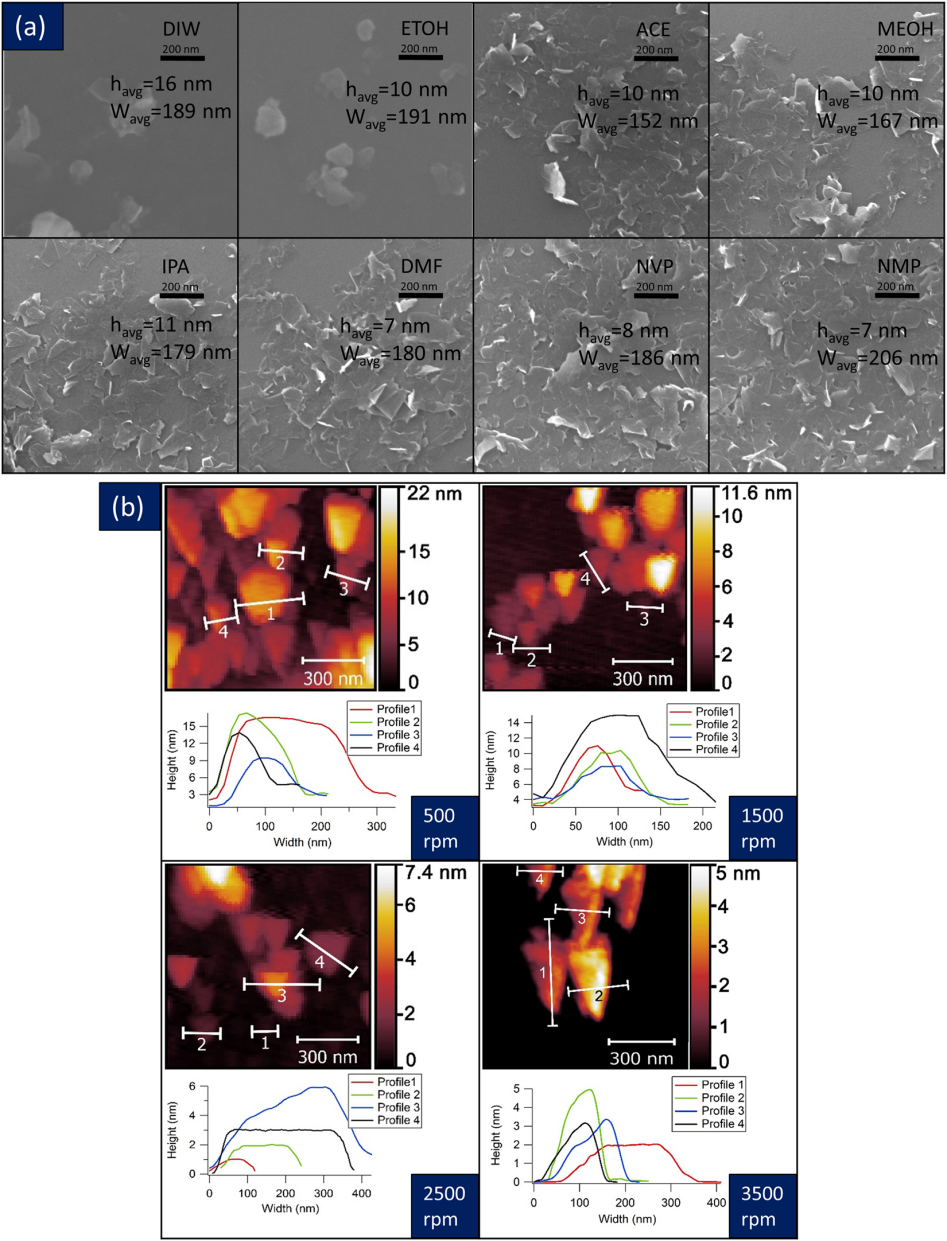
SEM and AFM Imaging of Exfoliated  WS_2_ Flakes. (**a**) SEM images of exfoliated WS_2_ flakes in different solvents; (**b**) AFM images of exfoliated WS_2_ flakes that were centrifuged at different speeds. Centrifugation time for SEM and AFM images were 90 minutes.

Scanning Electron Microscopy (SEM) and Atomic Force Microscopy (AFM) was conducted on all the exfoliated samples. Figure 3(a) presents the SEM image panel of the flakes exfoliated from all the solvents. The average height and width of the flakes are presented in the image. The average height and width of the flakes were calculated based on the statistical data obtained from 10 different samples, and over 100 flakes for each sample and these results are shown in detail in Figure S2 (Suppl.). NMP and DMF gave the smallest average height of ~7 nm. The height value may represent ten layers in average, but over 60% of these flakes had a height less than 5 nm, indicating 3–6 layers.

Figure 3(b) presents the AFM height profiles from several flakes. In Fig. 3(b), striking images of the triangular shape of the WS_2_ few layers are noted in the images. All these samples were exfoliated in DMF for 40 hours and had the same exfoliation condition. Such triangular shape confirms the lattice structure of WS_2_ as presented in the schematic of Fig. 1. The height image in each AFM micrograph shows the number of layers. It is clear that these are few layer flakes anywhere from 1–10 layers. In Figure S3 (Suppl.), the width of the flakes and the number of layers of the flakes exfoliated in DMF that were centrifuged at different speeds, are presented as histograms from the AFM images. We found that NMP is the best solvent with respect to both width and few number of layers of the flakes followed by NVP, DMF, IPA, MeOH, Ace, EtOH, and H_2_O. The ideal solvent should produce few number of layers with larger width of the 2D crystal. Slight differences in exfoliation yield are seen among different solvents, but the overall trends remain the same. Summarizing the gathered data using UV-visible spectra (Fig. 2), AFM statistics and SEM (Fig. 3), it can be concluded that as the wavelength of the major peak around 630 nm is decreased, the solution has a better yield regarding having a fewer number of layers. Also as the average width of the flakes inside the solution is increased, the scattering component of the UV-visible spectra is increased, and the slope of that component in logarithmic presentation (Fig. 2b) is larger.

Centrifugation resulted in exfoliated layers being separated from the bulk particles. We studied the effect of centrifugation speed on the shift in the wavelength of the WS_2_ signature peak obtained using UV-Visible optical absorbance as presented in Figure S4 (Suppl.). Samples that were exfoliated but not centrifuged were studied and compared to samples that were exfoliated and centrifuged at different speeds for 90 minutes. At 500 rpm (230 G), we saw the greatest change in the blue shifting of the optical absorbance to lower wavelengths. This suggests isolation of the exfoliated layers is highest after centrifugation. The absorbance was blue shifted to 640 nm (1.96 eV) suggesting isolation of few layers in solution. Above 500 rpm, the blue shift in the optical absorbance was small. It is observed that the width and height of flakes had a huge difference when the centrifugation speed was over 1500 rpm. However, when the centrifugation speed was increased to 3500 rpm, there was not a significant difference in the height of flakes while the width of flakes was reduced.

Finally, we also studied the effect of temperature on the exfoliation of the sample in DMF presented in Figure S5 (Suppl.). The sonication bath was heated to different temperatures from 30 °C to 60 °C during exfoliation and all of these samples were exfoliated in DMF. At low temperatures between 30–50 °C, there is no increase in UV-Visible optical absorbance suggesting no change in exfoliation yield with temperature. However, when the temperature was increased to 60 °C, it is striking to see an impressive increase in optical absorbance. The increase in optical absorption is a result of increasing number of few layers with temperature in the DMF solution. Past reports on graphene have reported a temperature of 127 °C-2000 °C to get appreciable few layers in thermal exfoliation process^48^. We see an unambiguous change in exfoliation yield at these low temperatures suggesting temperature dependent exfoliation can be a useful method for nanomanufacturing of TMDs. For 10°C increase in temperature above 50 °C, there is 300% increase in optical absorbance suggesting an increase in exfoliation yield by a similar amount. The mechanism of such thermal exfoliation in DMF and other solvents needs to be further understood in the future and beyond the scope of this paper.

**Figure 4:**
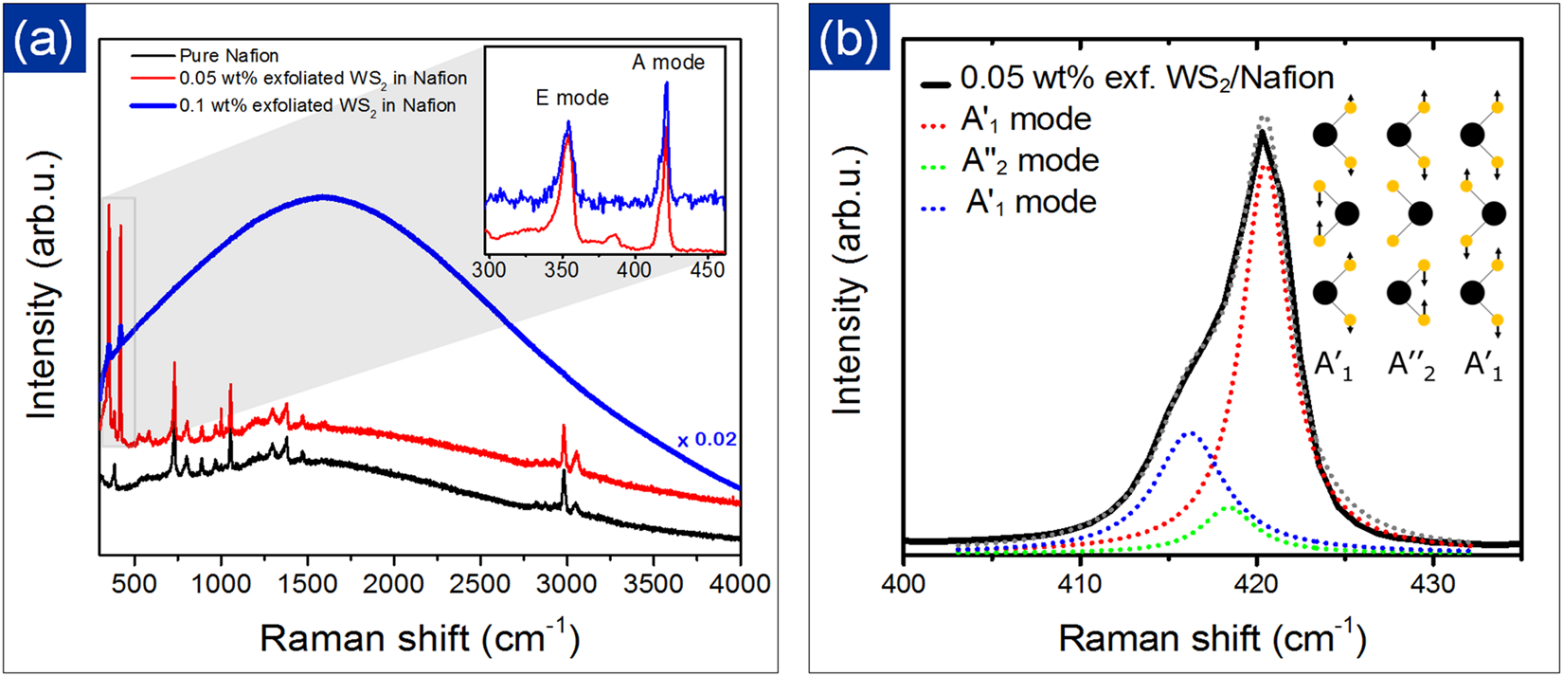
Raman Spectroscopy: (**a**) Resonant Raman spectra of pure Nafion, 0.05 wt.% and 0.1 wt. % exfoliated WS_2_ in Nafion suggesting photoluminescence in 0.1 wt.% exfoliated WS_2_-Nafion nanocomposite; the inset shows the deconvoluted spectra of both exfoliated WS_2_ sample. (**b**) The fitting of A mode spectral region in exfoliated sample confirmed the 3 L structure of WS_2_ inside the nanocomposite suggesting a high dispersion of 3 layer flakes in the composite. The excitation wavelength was 633 nm.

Resonant Raman measurements provide fundamental information on the vibrational modes and internal structure of the 2D layered material. One can investigate fine details of lattice interactions from resonant Raman spectra due to strong electron-phonon interactions and appearance of the second-order Raman peaks. Due to similar energy bandgap and close energy of the A excitonic resonance, the 633 nm laser excitation also leads to resonant Raman signals in WS_2_. Figure 4(a) shows the unpolarized 633 nm Raman spectra of our pure Nafion, 0.05 wt.% and 0.1 wt. % exfoliated WS_2_ in Nafion samples. The inset shows the photoluminescence of the 0.1 wt.% WS_2_-Nafion composites. The intriguing light emission in these nanocomposites suggest access to the electronic states within the nanocomposites and suggest excellent dispersion of few layers. For a low concentration of WS_2_, Raman lines of Nafion are well visible, whereas, for higher concentration, the luminescence of WS_2_ dominates the recorded spectrum suggesting these composites themselves are good candidates to study photoluminescence. A pure Nafion spectrum was also measured and used for deconvolution of spectra obtained for 0.05 wt. % exfoliated WS_2_ sample. 2H-WS_2_, like most TMDs, has the D^4^_6h_ point group symmetry and 18 vibrational modes in the Γ-point, out of which, E^2^_2g_, E^1^_2g_, E^1^_g_, and A_1g_, are active first-order Raman modes^28,47^. Usually, E^1^_2g_ and A_1g_ are only observed in Raman spectrum, as the E^2^_2g_ has a too low frequency for typical experimental set-ups, and the E^2^_2g_ is forbidden in backscattering geometry. Additionally, under resonant conditions, second-order Raman peaks is also observed. Resonant Raman spectra of our WS_2_ composite samples contain the second-order 2LA (M) peak at 351 cm^−1^ in addition to the first-order E^1^_2g_(Γ), and the out-of-plane A modes at 355, and ~420 cm^−1^, respectively. For 633 nm excitations, the 2LA(M) mode is significantly enhanced with respect to the E^1^_2g_ while being in resonance with the excitonic absorption peaks. The lines positions are the same for both WS_2_ samples. Besides, both samples show a broadening and asymmetric line shape of the out-of-plane A mode, indicating a few layer flake morphology of WS_2_^46,49,50^. In order to estimate the number of layers of WS_2_ inside the nanocomposite, we analyzed the line shape of the A mode (Fig. 4 (b)). For few layer samples, the symmetry (and the number of vibrational modes) of the WS_2_ structure is reduced to the D_3h_ and D_3d_ point groups for an odd and even number of layers, respectively. As shown recently, for a few layer WS_2_, the A mode peak consists of several components, and their number equals to the number of layers^49^. For an odd number of layers all components represent Raman active modes, i.e., both, in- and out-of-phase vibrations, are Raman active. On the other hand, for samples with an even number of layers, only in-phase vibrations are Raman active, but they are accompanied by weak infrared active modes located in between Raman modes. Peak deconvolution using pure Lorentzian lines of the A mode peak in 0.05 wt. % exfoliated WS_2_ yields undoubtedly three components. Moreover, we were able to get a very good fit using three components located at 420.5 cm^−1^, 418.5 cm^−1^ and 416.2 cm^−1^, respectively, i.e., at the locations reported previously for three layer WS_2_^49^. Out of these three component modes, the first and last ones possess the ${A}_{1}^{{\prime} }$ symmetry (in-phase vibration), whereas the middle mode originates from an infrared active ${A}_{2}^{{\prime\prime}}$ mode, characterized by the middle layer fixed and the sulfur atoms (top and bottom layers) vibrating out-of-phase. The performed fitting indicates clearly that our WS_2_-Nafion composite samples contain mostly 3 L WS_2_ flakes. These observations are in-line with past reports on determining a number of layers based on Raman spectroscopy^49^.

**Figure 5:**
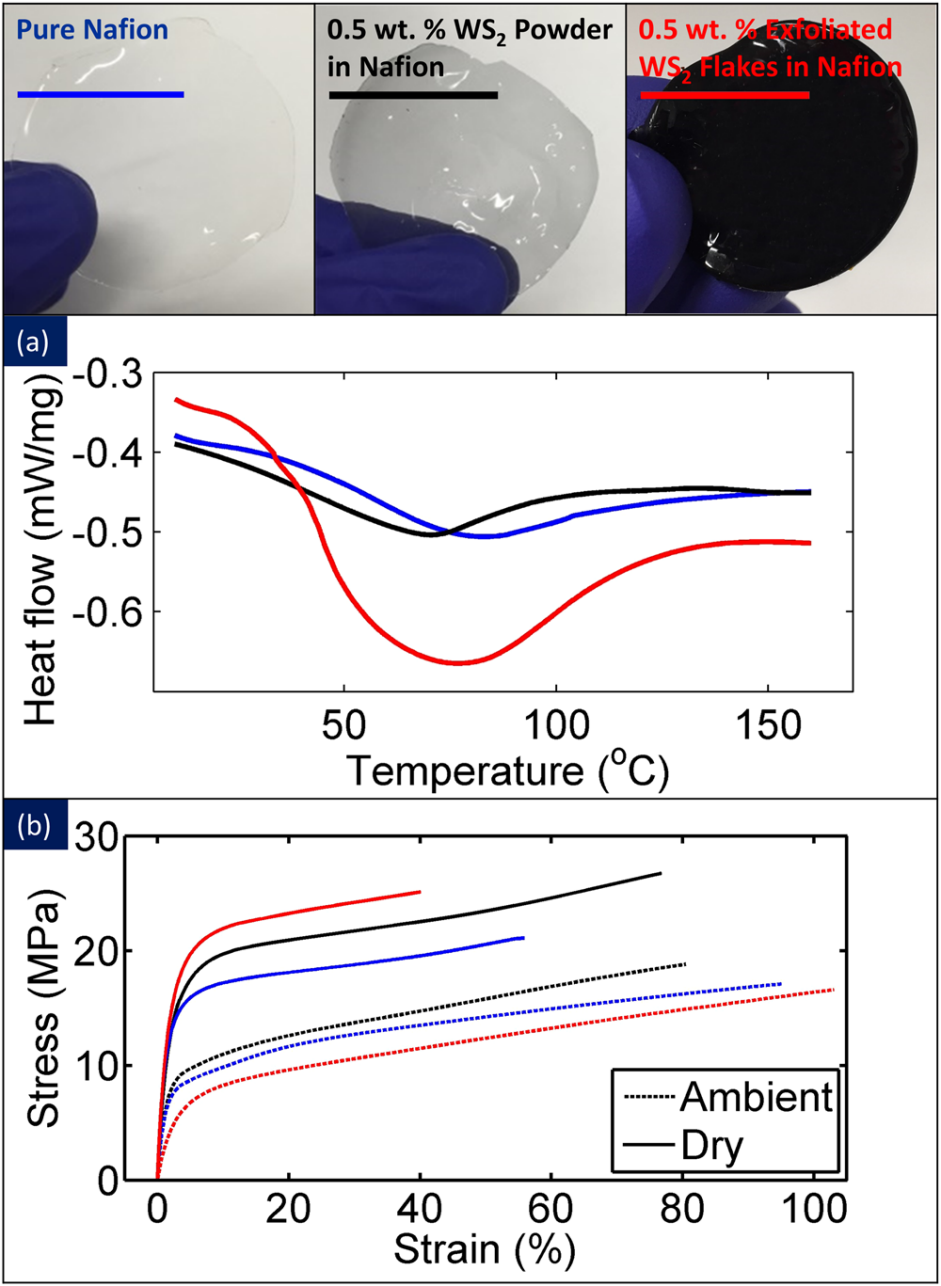
Differential Scanning Calorimetry and Dynamic Mechanical Analysis: (**a**) Thermograph of fabricated Nafion composites and the valley shows the evaporation of the water from the samples, (**b**) Results of tensile test on Nafion composites in ambient and dry conditions suggesting softening of the WS_2_ Nafion actuators in wet condition. In the dry condition, they showed 114% improvement in elastic modulus with the addition of 0.5 wt.% WS_2_.

Differential scanning calorimetry was used to investigate the water uptake in these nanocomposites. Figure 5(a) presents the thermograph of the pure Nafion, 0.5 wt. % WS_2_ powder in Nafion, and 0.5 wt. % exfoliated WS_2_ flakes in Nafion composites for the ambient condition. The large peak around 80 °C indicates the evaporation of water inside the actuators. When the same temperature range was applied for the second time immediately after the first run, the peak does not present itself anymore confirming water uptake and its evaporation (Figure S6(suppl.)). As it is observed in Fig. 5(a), the peak containing 0.5 wt. % exfoliated WS_2_ flakes is the largest, and it showed the highest water content among the samples. These results show higher porosity of Nafion composites that contain the exfoliated WS_2_ flakes compared to pure Nafion and Nafion-WS_2_ powder based nanocomposite. Figure S7(suppl.) compares the thermograph of the same three samples in fully wet condition. The peak amplitude is higher for all of the samples in comparison to their ambient condition suggesting water is the reason for the large dip in the endotherm. What is striking is the peak value of Nafion composite with exfoliated WS_2_ flakes is 300% larger than the pure and powder samples. We also calculated the first and second sweeps thermographs for ambient condition samples to calculate the water evaporation enthalpies. The enthalpies of evaporation ΔH (pure Nafion) = 11.056 mJ/mg; ΔH (0.5 wt.% powder WS_2_ in Nafion) = 13.2823 mJ/mg and ΔH (0.5 wt.% exfoliated WS_2_ in Nafion) = 42.107 mJ/mg. These suggest, greater than three times increase in enthalpy of water evaporation for the nanocomposites of exfoliated WS_2_/Nafion compared to pure Nafion. The exfoliated WS_2_-Nafion composite is, therefore, superb in water uptake ideal for proton exchange membranes for fuel cells.

Next, mechanical properties of the samples were investigated using Dynamic Mechanical Analysis (DMA). Figure 5(b) compares the results of a tensile test of the pure Nafion, 0.5 wt. % WS_2_ powder in Nafion, and 0.5 wt. % exfoliated WS_2_ flakes in Nafion composites at the ambient and dry condition. This plot shows that the sample containing 0.5 wt. % exfoliated WS_2_ flakes has the least yield strength and elastic modulus at ambient condition. The average measured values were 159.5 MPa for pure Nafion, 165.1 MPa for 0.2 wt.% bulk WS_2_ powder-Nafion nanocomposite, 157.6 MPa for 0.05 wt.% exfoliated WS_2_-Nafion nanocomposite, 152.8 MPa for 0.1 wt.% exfoliated WS_2_-Nafion nanocomposite, 148.4 MPa for 0.2 wt.% exfoliated WS_2_–Nafion nanocomposite and 140.7 MPa for 0.5 wt.% exfoliated WS_2_-Nafion nanocomposites. As the amount of exfoliated WS_2_ increases, the mechanical properties decrease gradually suggesting increased water content in the sample with the addition of exfoliated WS_2_ in wet condition. However, the tensile test data on dry samples showed the highest strength was achieved for 0.5 wt.% exfoliated WS_2_-Nafion nanocomposite, which shows good dispersion. The measured values were 228.6 MPa for pure Nafion, 211.7 MPa for WS_2_ powder Nafion nanocomposite, 217.6 MPa for 0.05 wt.% exfoliated WS_2_-Nafion nanocomposite, 221.9 MPa for 0.1 wt.% exfoliated WS_2_-Nafion nanocomposite, 235.0 MPa for 0.2 wt.% exfoliated WS_2_-Nafion nanocomposite and finally 261.5 MPa for 0.5 wt.% WS_2_-Nafion nanocomposite. This suggests that water has significant activity in determining the mechanical properties of these nanocomposites even for pure Nafion. An increase in elastic modulus of dry samples by 185% compared to the wet samples for the 0.5 wt.% exfoliated samples suggest strong water adsorption in the exfoliated samples. In Nafion-graphene nanocomposites, addition of 0.1 wt.% and 1 wt.% graphene decreased the water uptake by twice^15^. In contrast, the water uptake increased when exfoliated WS_2_ was added to the Nafion. The measured values were 16.6% for pure Nafion, 16.8% for 0.2 wt.% WS_2_ powder-Nafion Nanocomposite, 17.1% for 0.05 wt.% exfoliated WS_2_-Nafion nanocomposite, 17.9% for 0.1 wt.% exfoliated WS_2_-Nafion nanocomposite, 20.5% for 0.2 wt.% exfoliated WS_2_-Nafion nanocomposite and 22.2% for 0.5 wt.% exfoliated WS_2_-Nafion nanocomposite. These values were calculated using this equation:

1 $$Water\,uptake\,( \% )=\frac{{m}_{ambient}-{m}_{dry}}{{m}_{wet}-{m}_{dry}}$$

where m_ambient_ and m_wet_ are the mass of Nafion composite that was kept in ambient and in water bath for 48 hours, respectively. m_dry_ is the mass of Nafion composite that was kept at 110 °C for 12 hours.

These results can be another indication of water content inside exfoliated sample in comparison to bulk and pure sample. Recent work has shown that clean non-aged TMDs such as MoS_2_ and WS_2_ are hydrophilic atomic layers^51^. Liquid exfoliated WS_2_ nanosheets have recently been demonstrated as ultrasensitive and stable chemiresistive humidity sensors^52^. Even single-layer of WS_2_ or MoS_2_ is a composite of a relatively more hydrophilic metal and a relatively less hydrophilic chalcogen atom^51^. Sonication and processing can enable the replacement of the chalcogens by oxygen from the ambient (during recasting process) which likely occupy the sulfur vacancy sites. Oxygen is a known hydrophilic atom due to its tendency to form hydrogen bonds with water molecules^53^. We believe this is the most likely mechanism for significantly increased water uptake in exfoliated samples and not seen in bulk WS_2_ powder based nanocomposites. The bulk powders were not sonicated before mixing, and thus no vacancies were created for oxygen atoms to occupy and bond with other water molecules subsequently. Another indication of higher porosity of the samples is the difference in their thickness while the same amount of Nafion was used. Table 3 compares the thickness of Nafion composites. The thickness of 0.5 wt. % exfoliated WS_2_ flakes in Nafion was 34% larger than 0.5 wt. % WS_2_ powder-Nafion composite and pure Nafion. Since the same amount of Nafion solution was used for all of the samples and the WS_2_ flakes have negligible volume, the higher thickness of the exfoliated WS_2_-Nafion composite can only be explained by water uptake by the samples (samples becoming fluffy due to increased water uptake) that contain exfoliated WS_2_ flakes.

**Table 3:** Properties of Nafion actuator samples.

	pure Nafion	0.2 wt% WS_2_ bulk in Nafion	0.05 wt% exfoliated WS_2_ in Nafion	0.1 wt% exfoliated WS_2_ in Nafion	0.2 wt% exfoliated WS_2_ in Nafion	0.5 wt% exfoliated WS_2_ in Nafion
E_ambient_ (MPa)	159.5	165.1	157.6	152.8	148.4	140.7
E_dry_ (MPa)	228.6	211.7	217.6	221.9	235.0	261.5
Proton conductivity σ (S/cm)	0.0162	0.0163	0.0174	0.0185	0.0204	0.0260
Water uptake (%)	16.6	16.8	17.1	17.9	20.5	22.2
Thickness (µm)	70	70	74	78	86	93
Strain amplitude at 0.1 Hz (%)	0.0881	0.0899	0.0987	0.1102	0.1414	0.1537
Increase in strain amplitude (%)	Reference	1.8	11.8	24.8	60.5	74.1

**Figure 6:**
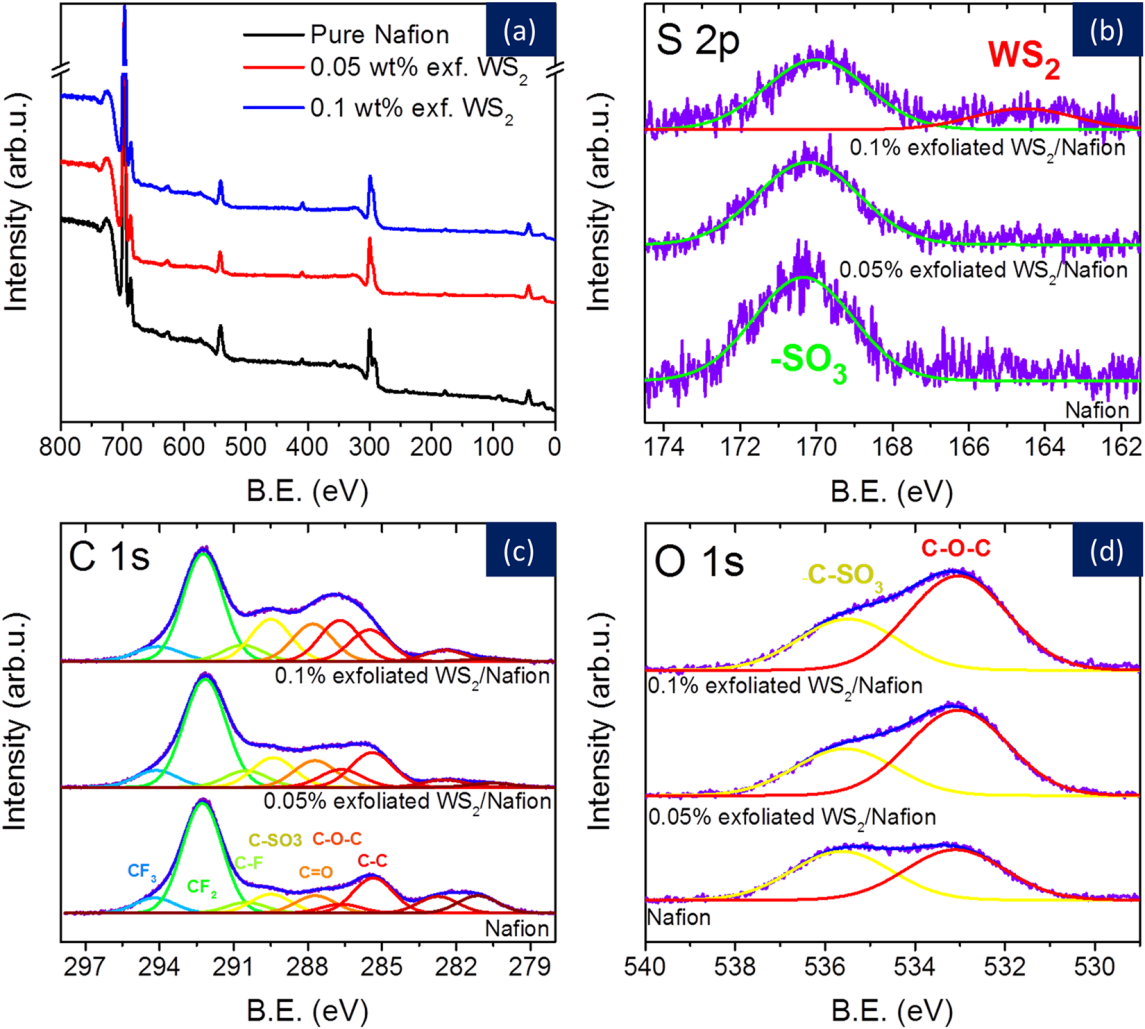
X-ray photoelectron spectroscopy of Nafion-TMD Nanocomposites: XPS analysis of pure Nafion, 0.05 wt.% and 0.1 wt. % exfoliated WS_2_ in Nafion showing a set of survey spectra, and high-resolution regions of C 1s, O 1s and S2p lines. (**a**) The complete spectra of all the three samples; (**b**) S 2p spectra showing -SO_3_ bonding in Nafion, and weak W-S bonding only for 0.1wt.% WS_2_ dispersed in Nafion; (**c**,**d**) C 1s and O 1s spectra for Nafion, 0.05 wt.% and 0.1 wt.% exfoliated WS_2_ flakes in Nafion. The improved C-SO_3_, C-O-C and C=O peaks in 0.1 wt.% suggest successful integration of WS_2_ flakes with the polymer matrix.

X-ray photoelectron spectroscopy (XPS) was used to analyze the surface of the sample and is presented in Fig. 6. The survey spectra and the high-resolution regions of C1s, O1s, and S2p lines were measured for each sample. First, the large fluoride signal and the sulfur signal in the wide scan spectrum (Fig. 6a and b) indicates the success of incorporating Nafion chains to TMDs. The analysis shows for instance that the O1s peak consists of two dominant components, one at 533 eV and the other at 535.5 eV which were attributed to the C-O-C and C-SO_3_ oxygen bonding in the Nafion structure. The fitting of C1s region reveals several lines; each corresponds to the bonding in Nafion polymer structure. The lines at 285.4, 286.7, 287.7, 289.5, 290.5, 292.2, and 294 eV depict: C-C (sp3), C-O-C, C=O, C-SO_3_, C-F, CF_2_, and CF_3_ bonds, respectively. These peak assignments correspond to the Nafion chains. Interestingly, as the WS_2_ concentration increases so does the C-O-C signal. Also, for the 0.1 wt. % exfoliated WS_2_ sample; it is possible to observe the S2p line at 164.1 eV corresponding to W-S bonding in line with other reports^54^. The major line in this region (169.6 eV), visible for all three samples, originates from the C-SO_3_ bond in the Nafion structure.

**Figure 7:**
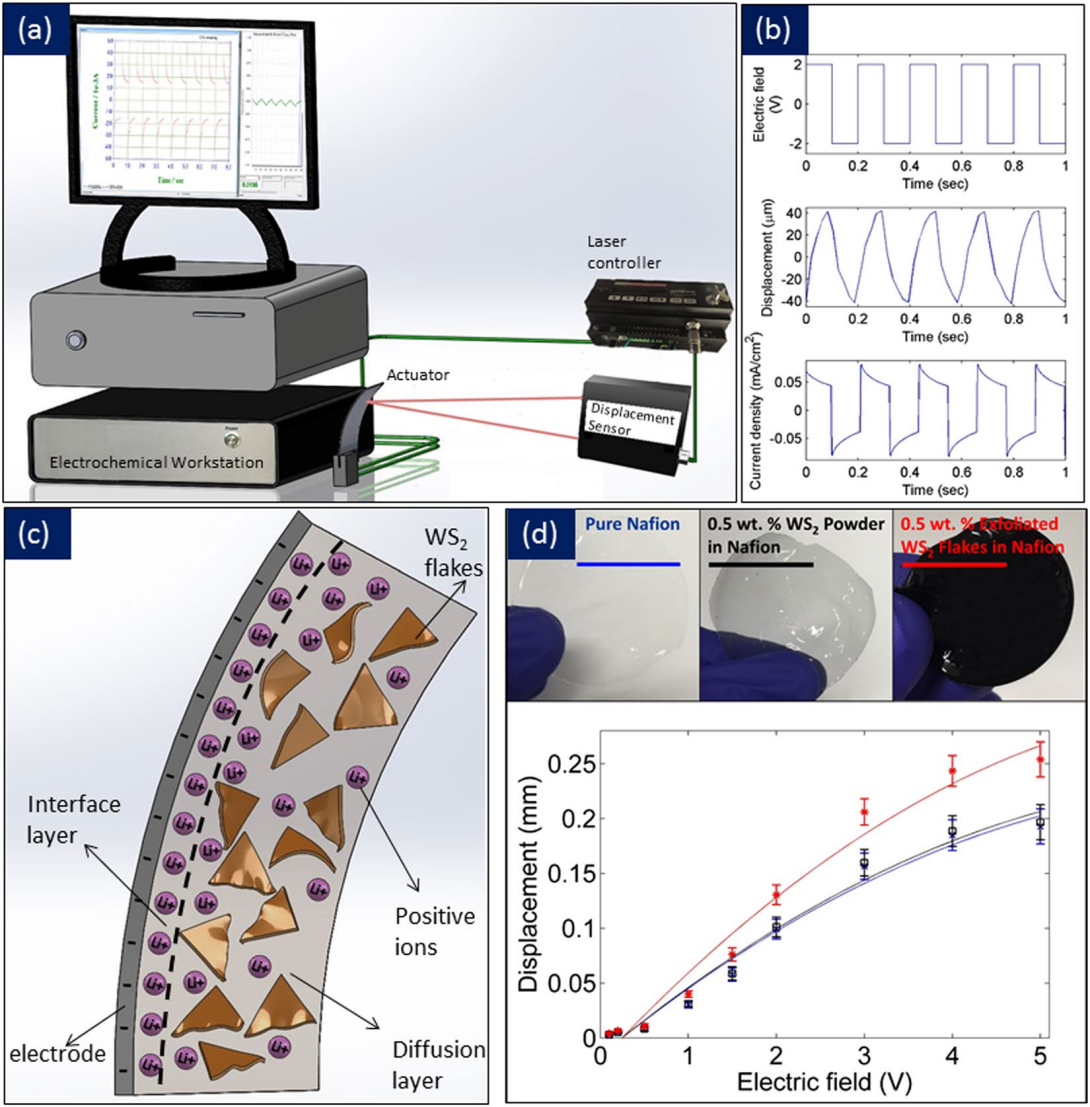
Testing Set-up, Actuation, and Actuator Model: (**a**) Schematic of the setup employed for characterizing the actuation of Nafion composites; (**b**) The actuation results of 0.5 wt. % exfoliated WS_2_ in Nafion composite at 5 Hz; (**c**) Schematic of different layers inside WS_2_ – Nafion composites suggesting electrode layer, interface layer and diffusion layer; (**d**) Displacement versus electric field for the different types of actuators at 1 Hz color coded in red, black and blue.

To understand the actuation mechanisms, we conducted testing the samples with a known electric field. Figure 7(a) presents the schematic of the electromechanical actuation set-up and testing. WS_2_-Nafion composites were cut into 13 mm × 5 mm, and their electro-mechanical tip displacements were studied. Figure 7(b) shows the captured signal during the actuation of 0.5 wt. % exfoliated WS_2_ flakes in Nafion at 2 V and 5 Hz operation. The electric field of 2 V was applied as a square signal while displacement and current were measured simultaneously. An electrochemical workstation was used for the excitation signal and the current feedback through two wires that are connected to the electrodes at both sides of the actuator. The displacement at the tip of the actuator was captured using the laser displacement laser as presented in Fig. 7(b). Figure 7(c) presents the schematic of the actuator cross-section excited by an electric field. Positive charges diffuse toward the negative electrode. Three following regions can be identified: electrode layer, an interface layer, and a diffusion layer. The electrode is the conductive layer on top. The diffusion layer conducts the ions inside the composites toward the electrode and vice versa. The interface layer is between the electrode and diffusion layer and contains a high concentration of positive ions (cations). The accumulation of ions at the surface of the composite causes the elongation of the surface and deformation of the actuator. For large deformations, considering constant curvature, the maximum strain at the surface of the actuator is given by the following equation:

2 $${\varepsilon }_{max}=\frac{2t\delta }{{L}^{2}+{\delta }^{2}}$$

where t is the thickness, δ the lateral displacement measured using a laser displacement sensor, and L is the free length of the actuator. Figure 7(d) presents the displacements versus electric field from 0.1 V to 5 V. As it can be observed, the displacements increased with the electric field in all the actuators. The 0.5 wt.% exfoliated WS_2_ actuators showed the largest displacement at all electric fields. These are quite intriguing that small amount of TMDs can produce a robust and enhanced mechanical response at low voltages. The actuation was almost twice as high for the exfoliated actuators compared to pure Nafion, and the tip displacements (~0.15 mm at 2 V for 10 mm actuator free length) are higher than nanotube^14^ and graphene-based actuators^15^ suggesting hydrophilic TMDs are excellent choices for Nafion-based actuators. In carbon nanotube-Nafion actuators, the experiments were performed by dipping the bimorph in LiCl solution whereas here we performed the experiments in air under an ambient condition with actuation caused by cation motion inside the actuator. Further, the amount of nanotube used was also high (18 w/w%) compared to the actuator performance^14^. Here, we have used minute loading of ~0.5 wt.% for significant improvement in actuation. In electro-active graphene actuator, the tip displacement of 0.15 mm at 0.5 Hz for actuator free lengths of 25 mm^15^ is actually smaller than our actuators. For an actuator length of 13 mm and since 3 mm was used for clamping the wires, the actuation occurs in 10 mm length of our samples. Thus for 10 mm actuator free lengths, the tip displacement of our actuator is 0.15 mm. For the same free length, we expect our actuators to perform 2.5 times better than graphene-based actuators. There are several reasons for this: 1) Addition of graphene and carbon nanotubes reduces the water content inside the actuators, which could potentially be a problem for long-term stability and operation as both nanotubes and graphene are hydrophobic materials and can lead to faster dehydration^15^; 2) TMDs are hydrophilic materials and thus can provide long term operation without drifts as these actuators by holding more water. Our results are quite consistent from this point from thickness measurements, a large dip in the differential scanning calorimetry and measurements using dynamic mechanical analysis all point to water in the exfoliated TMD actuators. Further, the increase in proton conductivity with increase in exfoliated TMDs definitely point to better working of these actuators and faster actuation as new proton conducting pathways being established with the addition of TMDs. The photoluminescence in our actuators is also a new addition not possible in graphene-Nafion actuators and thus brings added functionality to Nafion-based actuators.

A supplementary video shows the displacement of the actuator under positive and negative electric field. This video shows the large actuation of 0.5 wt. % exfoliated WS_2_ in Nafion sample right after doping and deposition of the silver electrode. The voltage was swept between −10 volts to +10 volts while the scanning rate was maintained at 1 V/s for this video. Grid lines behind the actuator were set up to follow the displacement of the tip. Each of the grid line squares behind the actuator is 1 mm wide. As the video shows, large bending displacements can be obtained in the exfoliated sample. The sample is seen to follow the electric field, and the actuation is fully reversible. Due to non-symmetric plating, the actuator is also seen to undergo torsion during the positive half cycle. As the polarity of the electric field changes in the actuator so does the motion suggesting direct evidence for Li^+^ ion (cation) induced motion in this electro-mechanical actuator. This unexpected and large actuation in the video is a result of complex effects from the electrode symmetry, internal strains due to doping, water uptake, electro-osmotic pressure and recasting process, all of which can have a profound effect on actuation. In the video, one can see the fast bending of the actuator when the field is applied followed by a slow relaxation when the polarity is changed. This bending motion is a result of the movement of the cations due to the electro-osmotic pressure and diffusion of water through the nanochannels^15^. A reinforcing filler that can block or delay the water migration will be advantageous in improving the overall actuator performance, and thus 2D materials such as graphene and TMDs can provide a highly tortuous path to delay the onset of this water migration^15^. One could potentially obtain twisting, rolling, torsion and non-symmetric bending deformation in these actuators by changing the symmetry of the metal plating, ion density, water uptake and electric field.

**Figure 8:**
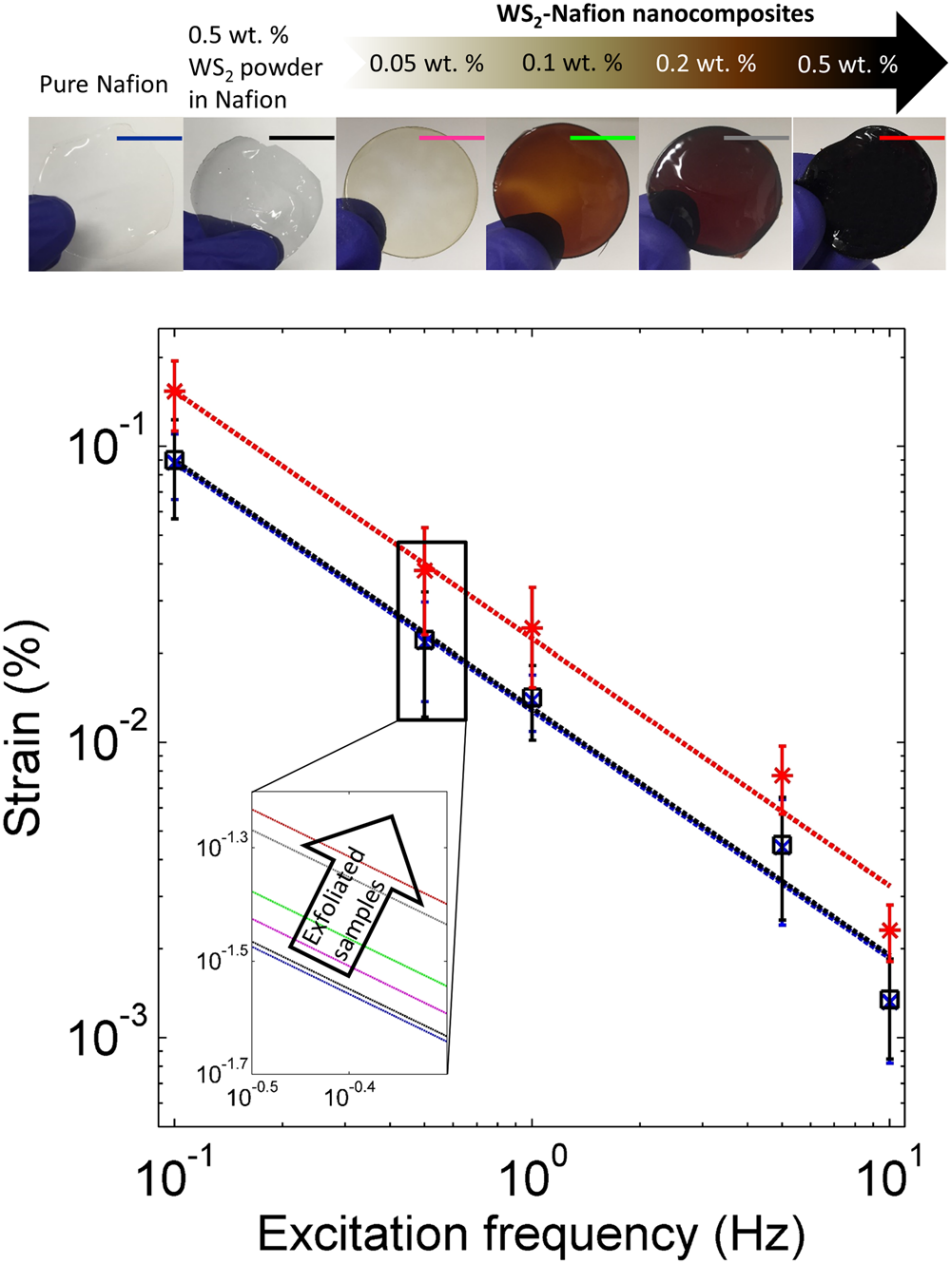
Strain Amplitudes Vs. Excitation Frequency: Actuation strains of fabricated samples at different excitation frequencies. The exfoliated WS_2_-Nafion nanocomposites resulted in better actuation at all frequencies tested. The samples are color coded in strain versus excitation frequency graph.

**Figure 9:**
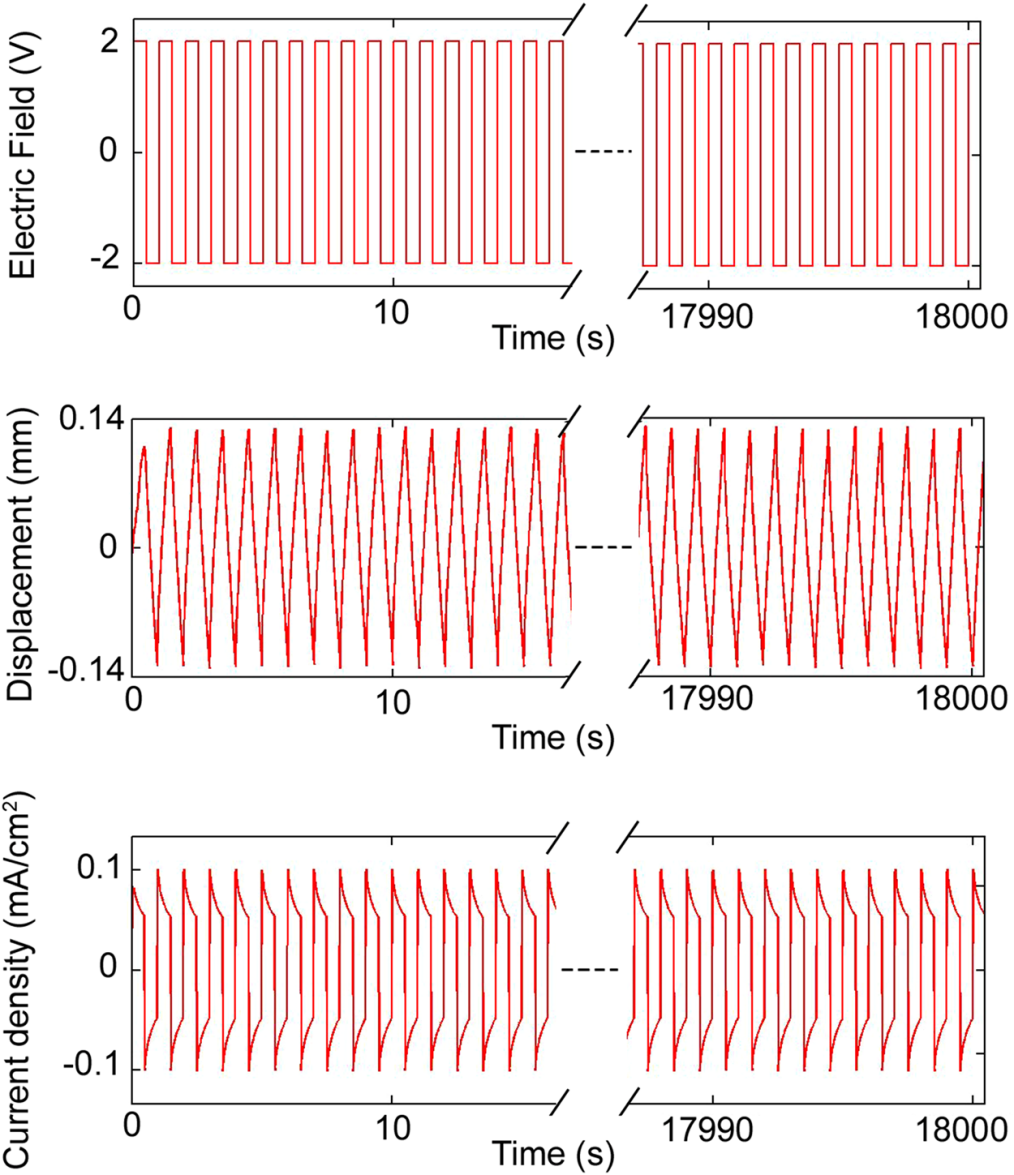
Long Term Actuation: Actuation of ~0.5 wt. % exfoliated WS_2_-Nafion composite shows consistent behavior over 18000 seconds suggesting excellent stability of these systems.

Figure 8 presents the optical images of the six different composites that were investigated in this research. The top image from left to right are pure Nafion actuator, ~0.5 wt. % WS_2_ powder in Nafion, ~0.05 wt. % exfoliated WS_2_ flakes in Nafion, ~0.1 wt. % exfoliated WS_2_ flakes in Nafion, ~0.2 wt. % exfoliated WS_2_ flakes in Nafion and ~0.5 wt. % exfoliated WS_2_ flakes in Nafion. One can see the exfoliated samples changing colors as the amount of WS_2_ increases turning almost dark at 0.5 wt.% WS_2_. These suggest excellent dispersion of the exfoliated WS_2_ in Nafion. Figure 8 presents the actuation of all the six samples. The electric field amplitude for all of these experiments was kept at 5 V. It was observed from Fig. 8 that adding the WS_2_ bulk powder to Nafion did not produce a significant difference in actuation compared to pure Nafion. However, by adding exfoliated WS_2_ flakes to Nafion, the actuation of the Nafion composite improves with increasing WS_2_ weight percentage. There is a significant difference between the actuation of 0.1 wt. % and 0.2 wt. % of exfoliated WS_2_-Nafion nanocomposites. The inset in Fig. 8 shows the increasing strains with small additions of WS_2_ flakes into the Nafion polymer. The actuation strain increased by ~74% at 1 Hz for 0.5 wt. % exfoliated WS_2_-Nafion nanocomposites in comparison to pure Nafion samples suggesting adding small amounts of WS_2_ flakes into Nafion improves its overall electro-mechanical actuation. As the frequency increases, the strains decrease. The strain versus frequency has a linear relationship even at these low concentrations of WS_2_ suggesting excellent dispersion and actuation. Figure S8 (Suppl.) presents the voltage sweep and strain amplitude developed. The radius of curvature and the strains are presented. The largest strain was calculated to be 0.27%. Table 3 further summarizes the actuation results for WS_2_-Nafion composites. The strain amplitudes were compared to the pure Nafion when presenting the results. Figure 9 presented the results of long-term continuous actuation tests (5 hours) on the 0.5 wt.% WS_2_ flakes-Nafion composite when the 2 V square wave was applied at 1 Hz. The results suggest no drift in the actuators over this period, voltage and frequency suggesting excellent stability of the WS_2_-Nafion composites.

**Figure 10:**
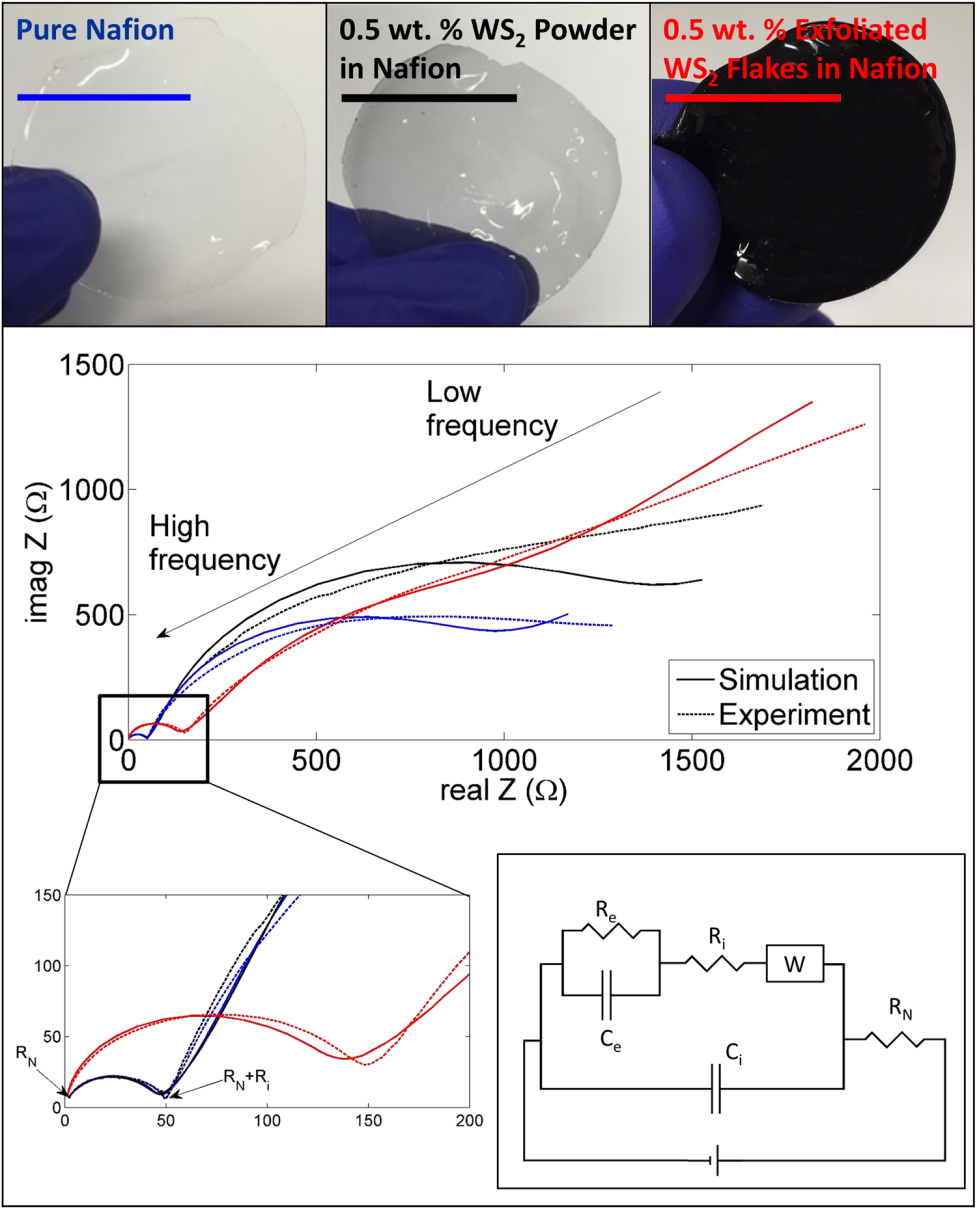
AC Impedance Spectroscopy: AC impedance spectroscopy based characterization of fabricated Nafion composites and its corresponding equivalent circuit. The excellent agreement between the experiments and simulations presented indicate the description of the actuator is accurate and can serve as a model to explain the electromechanical behavior of Nafion-TMD nanocomposite.

One of the important aspects of any electromechanical actuator is to explain the experimental results based on theory/simulations. Here we have presented an equivalent circuit model based on the different layers of the actuator. Figure 10 presents the equivalent circuit model of the WS_2_-Nafion composite actuator based on AC impedance spectroscopy. The R_N_ is the resistance of the Nafion as a solid electrolyte for the electrochemical system, and W is the Warburg resistance for the diffusion of the ions toward the negative electrode. R_i_ and C_i_ are the resistance and capacitance of the interface layer, respectively. R_e_ and C_e_ are the resistance and capacitance of the electrode, respectively. The excellent agreement between the experimental values and the simulation results based on the equivalent circuit suggest the circuit model presented explains well the working of this actuator. AC impedance spectroscopy was carried out in the range of 0.01 Hz to1 MHz, and the amplitude of excitation was 0.05 to have a linear response. Figure 10 shows the experimental data and the fitted curve based on the suggested equivalent circuit. This circuit was selected based on the internal structure of the actuator and the experimental data that was captured using AC impedance spectroscopy. According to the schematic structure of the Nafion in Fig. 7(c), R_e_ and C_e_ for electrode and R_i_ and C_i_ for the interface layer have been considered. For the diffusion layer, a Warburg element and R_N_ as the resistance of Nafion are placed in the circuit. If the R_e_ and C_e_ were removed, the circuit looks like the Randle’s circuit which has been used for many electrochemical systems^55^. Since the electrode is attached to the actuator in series, R_e_ and C_e_ as two parallel elements have been added to the Randle’s circuit. Based on the suggested equivalent circuit and experimental data, numerical optimization was employed to calculate the circuit elements value for each sample. The error for all of the simulated curve is less than 10% for all of the samples and considering the complex structure of Nafion; the model is precise, which agrees well with the experimental results. The high-frequency section of the Impedance curve is very similar to Randle’s circuit. As it has been shown, the end point of the curve is the resistance of the Nafion, and this value can be used to calculate the proton conductivity of the composite:

3 $$\sigma =\frac{t}{{{\rm{R}}}_{N}\times A}$$

where σ represents the proton conductivity, t is the thickness, R_N_ is the Nafion resistance, and A is the area covered by the electrodes. Other elements of the equivalent circuit are also summarized in Table 4. The proton conductivity was 160% higher for the exfoliated nanocomposite compared to pure Nafion samples and in line with literature reports^56^. As it is observed, the only resistance which is higher for the exfoliated sample in comparison to the pure sample is the interface resistance (R_i_). The other resistances including the Nafion resistance which represents the proton conductivity and Warburg element which represents the diffusion resistance of the composites are improved in the exfoliated samples significantly. Figure S9 (Suppl.) presents the cross-sectional view of the Nafion and 0.5 wt.% exfoliated WS_2_ Nafion nanocomposite sample under SEM. The cross-sectional SEM suggest two different morphologies. The pure Nafion is featureless (a), but the exfoliated WS_2_-Nafion nanocomposite show increased surface area with many features (b). In the figure, the flakes can be seen covered by the polymer suggesting uniform dispersion of the flakes. A single flake can be seen in the high magnification images in (c). The Nafion chains attached to the TMD flakes are providing proton conducting pathways for rapid proton transport through the sample. There is clear evidence for this as the proton conductivity steeply increases with increase in weight fraction of exfoliated TMDs as presented in Table 3. TMD-Nafion is also interconnecting the proton-conducting domains of the Nafion matrix thereby lengthening the proton-conducting channels of the sulfonic acid groups of the Nafion chain^56^. The increase in proton conductivity is also directly supported by the actuation results by almost twice.

**Table 4:** Equivalent circuit elements for Nafion actuators.

Element	pure Nafion	0.2 wt. % WS_2_ bulk in Nafion	0.05 wt. % exfoliated WS_2_ in Nafion	0.1 wt. % exfoliated WS_2_ in Nafion	0.2 wt. % exfoliated WS_2_ in Nafion	0.5 wt. % exfoliated WS_2_ in Nafion
R_N_ (Ω)	1.080	1.073	1.061	1.055	1.054	0.893
R_i_ (Ω)	42.9	41.8	49.2	77.0	84.6	125.6
W (mΩ)	2.0	1.8	1.5	0.8	0.8	0.7
C_i_ (µF)	0.3	0.3	0.241	0.240	0.243	0.250
R_e_ (Ω)	695.5	1019	660.1	644.5	583.7	439.3
C_e_ (mF)	0.22	0.23	0.29	0.34	0.36	0.28

In summary, the ability to convert electrical energy into mechanical motion is one of the fundamental building blocks for modern day actuators. The efforts in recent years have been towards understanding various nanomaterials such as carbon nanotubes and graphene in polymers. For the first time, we show interesting effects of incorporating TMDs such as WS_2_ in Nafion and demonstrate the improvement in the electromechanical actuation performance. The most common TMD namely MoS_2_ share similar structure as WS_2_ and are hydrophilic. This opens a broader field of applications of different types of TMDs (MoS_2_, WS_2_, MoTe_2_, etc.) for electrochemical or electromechanical actuation. Morphological studies of TMD–Nafion composites by SEM, AFM, XPS, DSC, and DMA show that highly exfoliated TMDs few layers are dispersed homogeneously in the Nafion polymer matrix by solvent recasting method, which is also confirmed through the photoluminescence of these nanocomposites. Improvements in Young’s modulus of 114% was observed with the addition of 0.5 wt.% of TMDs in dry condition. The proton conductivity increased 160% with the addition of 0.5 wt.% TMDs in Nafion suggesting TMDs improve proton conducting pathways in Nafion, whereas a considerable increase in water uptake (300%) was observed for exfoliated TMDs in Nafion. The increased water uptake due to hydrophilic WS_2_ additives made the actuator softer in wet and ambient conditions as seen in DMA measurements and is ideal for building Nafion-based actuators as these actuators need water for their working. The defective sites created in WS_2_ due to the knocking of some of the chalcogen atoms during sonication process provided binding sites for oxygen molecules from the ambient that has natural tendency to form bonds with water. Hence the increased water uptake by unexpected measures. Compared to the recast Nafion-based IPMC actuator, large strain amplitudes of ~0.15% were achieved in 0.5 wt.% exfoliated TMD based Nafion composite actuators. These actuators also showed unusually large bending displacements at large electric fields. The tip displacements were larger than graphene and nanotube-based actuators and used minute quantities of exfoliated TMDs. In the future, one can explore the use of thinner and smaller TMD flakes to fabricate actuators. This can improve the surface area to volume ratio and provide a highly tortuous path to delay the onset of the water migration that can improve the overall actuation performance. Other aspects of using thinner and smaller flakes include increase in interfacial resistance, improvement in proton conducting pathways, increase in photoluminescence and improved water uptake. The results thus provide a foundation for this rich area of study for future development of electromechanical actuators based on TMDs and in general for the development of shape changing materials based on polymers with reversibility. Since TMDs also have high optical absorption, such TMD-Nafion composites could also be prime candidates for creating stimuli-responsive composites, solar water splitting, proton exchange membranes and fuel cells.

## Methods

### Liquid Phase Exfoliation, Imaging, and Optical Absorption Spectroscopy

Two kinds of WS_2_ powder, micro powder with an average particles size of 0.4–1 µm and ultrafine powder with an average particles size of 90 nm were purchased from Graphene Supermarket. Solvents and Nafion® perfluorinated resin solution (5 wt. %) were purchased from Sigma-Aldrich. 5 mg/ml WS_2_ ultrafine powder obtained from Graphene Supermarket was sonicated in 100 ml glass vials in DMF inside the bath sonicator for 40 hours at maximum power (110 W), and then it was centrifuged at 1500 rpm (700 G) for 90 minutes. The number of layers of the WS_2_ flakes was characterized using a NaioAFM of Nanosurf in tapping mode configuration with a cantilever resonance frequency of ~148 kHz. HRTEM was conducted using a transmission electron microscope (TM) (model F20 FEI Tecnai) with an accelerating voltage of 200 kV. The sample was prepared by dropping drops of exfoliated WS_2_ in ethanol solution on lacey-carbon Cu grids (Ted Pella Inc., CA). The Cu grids were then dried in air to make sure there are sufficient isolated flakes to be observed. SEM images of flakes were obtained using a JEOL JSM-7000F instrument at 20 kV of power and under an ultra-high vacuum, 10^−5^ Pa. A secondary electron detector was utilized at 8 mm working distance to capture high-resolution images of the WS_2_ flake at magnifications as high as 100,000X.

The optical absorbance of the WS_2_ solutions and the nanocomposites were measured in a wavelength range of 300 nm-1100 nm using a Hitachi U–5100 ratio beam spectrophotometer. Raman spectroscopy was conducted in Horiba Xplora Raman system with the power of the laser was kept at 0.2 mW, and for calibration, the phonon mode from the silicon substrate at 520 cm^−1^ was used. The laser beam (λ_exc_ = 532 nm) was focused onto the surface using a 100 × objective. The power was much below one mW, not enough to induce heating in the samples.

### Sample Preparation

#### Nafion Actuator Fabrication

For pure Nafion actuator, 8 ml of Nafion was mixed with 4.6 ml DMF. The components were mixed in a 20 ml glass vial and sonicated for 1 hour. They were transferred to a glass petri dish with 48 mm in diameter. The dish was placed inside a vacuum oven, and it was heated at 110 °C for 4 hours followed by heating at 160 °C for 6 hours. After cooling down, the sample was immersed in DI water for 10 minutes; the as-casted film was then separated from petri dish by peeling. For drying the sample, the film was placed into a vacuum oven at 110 °C for 12 hours. After drying, the sample was soaked in 0.1 M lithium hydroxide at room temperature overnight for doping. After doping and drying in the ambient condition, both sides of the film were coated with thin film silver using sputtering (100 nm thickness). The sample was cut into the desired size (13 mm × 5 mm). The samples were then kept in the laboratory in ambient condition for 48 hours for relaxation before the actuation tests. The preparation steps are in line with many reports indicating that high-boiling-temperature polar solvents, N, N′-dimethyl formamide (DMF), ethylene glycol, hexamethyl phosphoramide, and dimethylacetamide, can be added to the Nafion solutions or used alone to prepare Nafion solutions to get good insoluble with desirable physical, and mechanical characteristics similar to those of the as received Nafion membranes^57^.

#### Bulk and Exfoliated WS_2_ actuators

For bulk WS_2_ actuator, the WS_2_ powder was mixed in DMF and Nafion solution. WS_2_ was exfoliated in DMF for these experiments using sonicator for 40 hours, followed by centrifugation at 1500 rpm to isolate the few layers. The concentration of the WS_2_ in DMF was 380 μg/ml. The weight of Nafion inside its initial solution was 43.7 mg/ml. Based on these numbers, the final solution for casting was prepared. For 0.5 wt. % exfoliated WS_2_ in Nafion, 4.6 ml of WS_2_ solution in DMF was added to 8 ml of Nafion solution. For other exfoliated samples the appropriate volume of WS_2_ solution in DMF was added to Nafion solution. In the end, the extra amount of pure DMF was added to all of the samples, so all of them had the same 4.6 ml of DMF. This is to ensure that the increase in actuation is not due to the amount of DMF. For example for 0.1 wt. % exfoliated WS_2_ in Nafion, 0.92 ml of WS_2_ solution in DMF was added to 8 ml of Nafion solution, and then 3.68 ml of DMF was added. The samples were then cast and prepared as mentioned above for pure Nafion actuator.

#### Resonant Raman Spectroscopy of Nanocomposites

Resonant Raman spectra of 0.05 wt. % and 0.1 wt. % exfoliated WS_2_ samples were recorded at room temperature in backscattering geometry with an inVia Renishaw micro-Raman spectrometer using a confocal setup with a 50x objective and excitation wavelengths of 633 nm. A silicon wafer was used for a calibration of the system. 1800 lines mm^−1^ grid was used to ensure a high spectral resolution of ~1 cm^−1^.

#### X-ray photoelectron (XPS) Spectroscopy

XPS measurements were carried out in VG Scientific MultiLab 3000 ultra-high vacuum surface analysis system equipped with CLAM4 hemispherical electron energy analyzer and X-ray source in the form of a dual-anode (Mg/Al) operating at 15 kV voltage and an emission current of 10 mA. XPS spectra were measured at the base chamber pressure in the 10^−9^ Torr range using a non-monochromatic Al Kα (hν = 1486.6 eV) X-ray radiation. To account for any possible sample charging, a C1s peak of the F-C-F bonding in Nafion at 292.2 eV was used for binding energy calibration. The analysis and deconvolution of XPS spectra were performed using the XPSPEAK41 software. The data was fitted using a pure Gaussian profile and a Shirley baseline subtraction.

#### Actuation Test Experiments

The actuator was clamped at one end, and the electric contacts are attached to the clamp as well. Micro-Trak laser displacement sensor was used to measure the deflection of the actuator. The laser was calibrated according to manufacturer specifications and then measured on the sample. Using CHI 660E electrochemical workstation, chronoamperometry and AC impedance spectroscopy tests were carried out on samples. For chronoamperometry test, a square waves electric field with different frequencies were applied on the electric contacts, and the current and displacement were measured using an electrochemical workstation and laser displacement sensor, respectively. For doing the AC impedance spectroscopy, measurements were carried out from 0.01 Hz to 1 MHz with the amplitude of 0.05 V for having a linear response. All samples were tested in identical conditions. The temperature was 23 °C with relative humidity RH~43%.

#### Dynamic Mechanical Analysis and Differential Scanning Calorimetry

Tensile test on the dry and hydrated samples at ambient condition was carried out using a Q800 TA Instruments Dynamic Mechanical Analyzer according to ASTM D882 standard. For measuring the water content of hydrated Nafion samples at fully wet and ambient conditions, Q20 TA Instruments Differential Scanning Calorimetry was used. A small piece of each sample was weighted and placed in a T_zero_ low mass pan, and the sample was heated from 10 °C to 180 °C.

## Electronic supplementary material

Supplementary

Supplementary video
