# Publisher Correction: Exfoliated WS_2_-Nafion Composite based Electromechanical Actuators

**DOI:** 10.1038/s41598-017-17924-8

**Published:** 2018-01-05

**Authors:** Masoud S. Loeian, Dominika A. Ziolkowska, Farhad Khosravi, Jacek B. Jasinski, Balaji Panchapakesan

**Affiliations:** 1 Small Systems Laboratory Department of Mechanical Engineering, Worcester Polytechnic Institute, Worcester, MA 01609 USA; 2 Conn Center for Renewable Energy Research, University of Louisville, Louisville, KY 40292 USA; 3 Faculty of Physics, University of Warsaw Pasteura 5, 02-093 Warsaw, Poland

Correction to: *Scientific Reports* https://doi.org/10.1038/s41598-017-14806-x, published online 03 November 2017

The original HTML version of this Article contained a typographical error in the publication date ‘03 November 2017’ which was incorrectly given as ‘06 November 2017’. This has now been corrected in the HTML version of the Article.

