# Publisher Correction: Exfoliated WS_2_-Nafion Composite based Electromechanical Actuators

**DOI:** 10.1038/s41598-018-22370-1

**Published:** 2018-03-06

**Authors:** Masoud S. Loeian, Dominika A. Ziolkowska, Farhad Khosravi, Jacek B. Jasinski, Balaji Panchapakesan

**Affiliations:** 1 Small Systems Laboratory Department of Mechanical Engineering, Worcester Polytechnic Institute, Worcester, MA 01609 USA; 2 Conn Center for Renewable Energy Research, University of Louisville, Louisville, KY 40292 USA; 3 Faculty of Physics, University of Warsaw Pasteura 5, 02-093 Warsaw, Poland

Correction to: *Scientific Reports* 10.1038/s41598-017-14806-x, published online 03 November 2017

An error occurred after publication resulting in the inadvertent omission of the Article and Volume numbers from the PDF version of this Article. This error has now been corrected in the PDF version of the Article; the HTML version was correct from the time of publication.

