# Supplementary material for: Exfoliated WS2-Nafion Composite based Electromechanical Actuators
Source: Sci Rep. 2017 Nov 3;7:14599. doi: 10.1038/s41598-017-14806-x (PMC5668253; doi:10.1038/s41598-017-14806-x)
Supplement: Supplementary file 1 — Supplementary [file 41598_2017_14806_MOESM1_ESM.doc]

**Exfoliated WS_2_-Nafion Composite based Electromechanical Actuators**

*Masoud S. Loeian^1^, Dominika A. Ziolkowska^2,3^, Farhad Khosravi^1^, Jacek B. Jasinski^2^ and Balaji Panchapakesan^1*^*

^1^*Small Systems Laboratory*

*Department of Mechanical Engineering*

*Worcester Polytechnic Institute*

*Worcester, MA 01609*

^2^*Conn Center for Renewable Energy Research*

*University of Louisville, Louisville, KY 40292*

^3^*Faculty of Physics, University of Warsaw*

*Pasteura 5, 02-093 Warsaw, Poland*

[**bpanchapakesan@wpi.edu*](mailto:*bpanchapakesan@wpi.edu)

Contents

[**Figure S_1_.** Absorption spectra of WS_2_ solutions in different solvents after removing the scattering component. 2](#__RefHeading___Toc480277383)

[**Figure S_2_.** AFM statistics on width and height of WS_2_ flakes for different exfoliation solvents. 3](#__RefHeading___Toc480277384)

[**Figure S_3_.** AFM statistics on width and height of WS_2_ flakes for different centrifuge speeds. 4](#__RefHeading___Toc480277385)

[**Figure S_4_.** Absorption of exfoliated WS_2_ solutions at DMF which are centrifuged with different speeds. 5](#__RefHeading___Toc480277386)

[**Figure S_5_.** Absorption of WS_2_ solutions at DMF exfoliated at different temperatures. 6](#__RefHeading___Toc480277387)

[**Figure S_6_.** Thermograph of WS_2_ –Nafion composites at ambient condition. 7](#__RefHeading___Toc480277388)

[**Figure S_7_.** Thermograph of the WS2- Nafion composites while they are fully wet. 8](#__RefHeading___Toc480277389)

[**Figure S_8_.** 3 frames of 0.5 wt. % exfoliated WS_2_ in Nafion composites at different voltage. 9](#__RefHeading___Toc480277390)

[**Figure S_9_.** SEM image of Nafion composites. 10](#__RefHeading___Toc480277391)


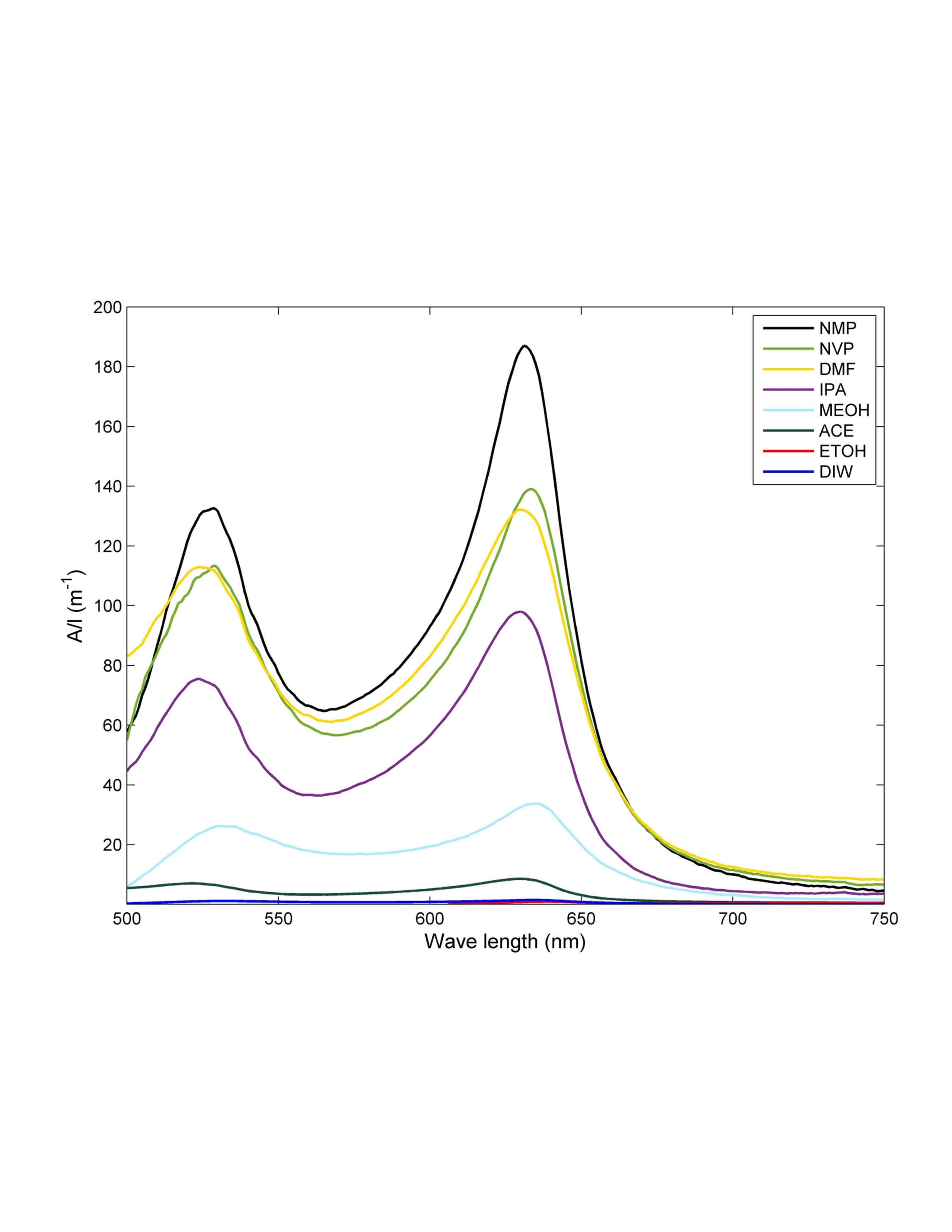


# **Figure S_1_.** Absorption spectra of WS_2_ solutions in different solvents after removing the scattering component.


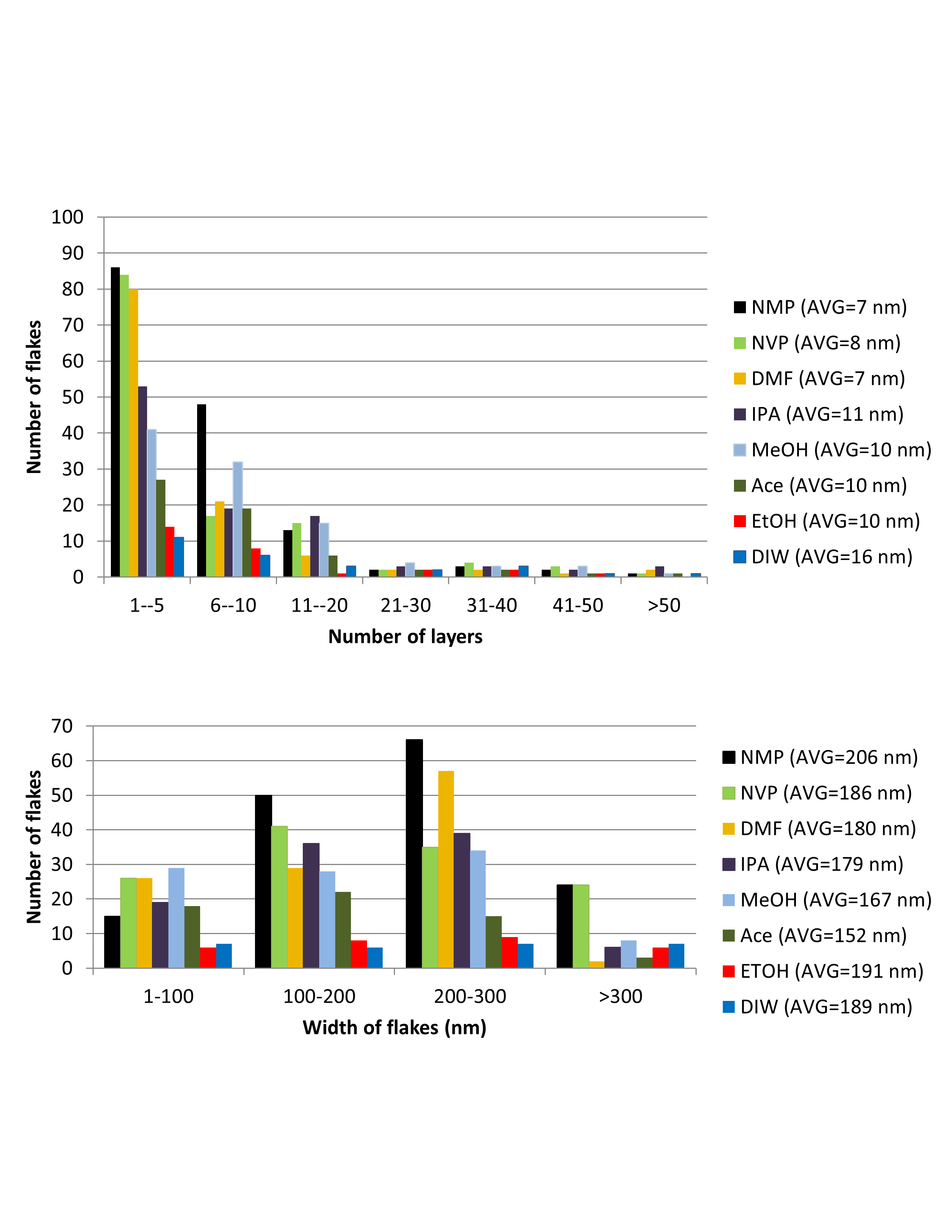


# **Figure S_2_.** AFM statistics on width and height of WS_2_ flakes for different exfoliation solvents.


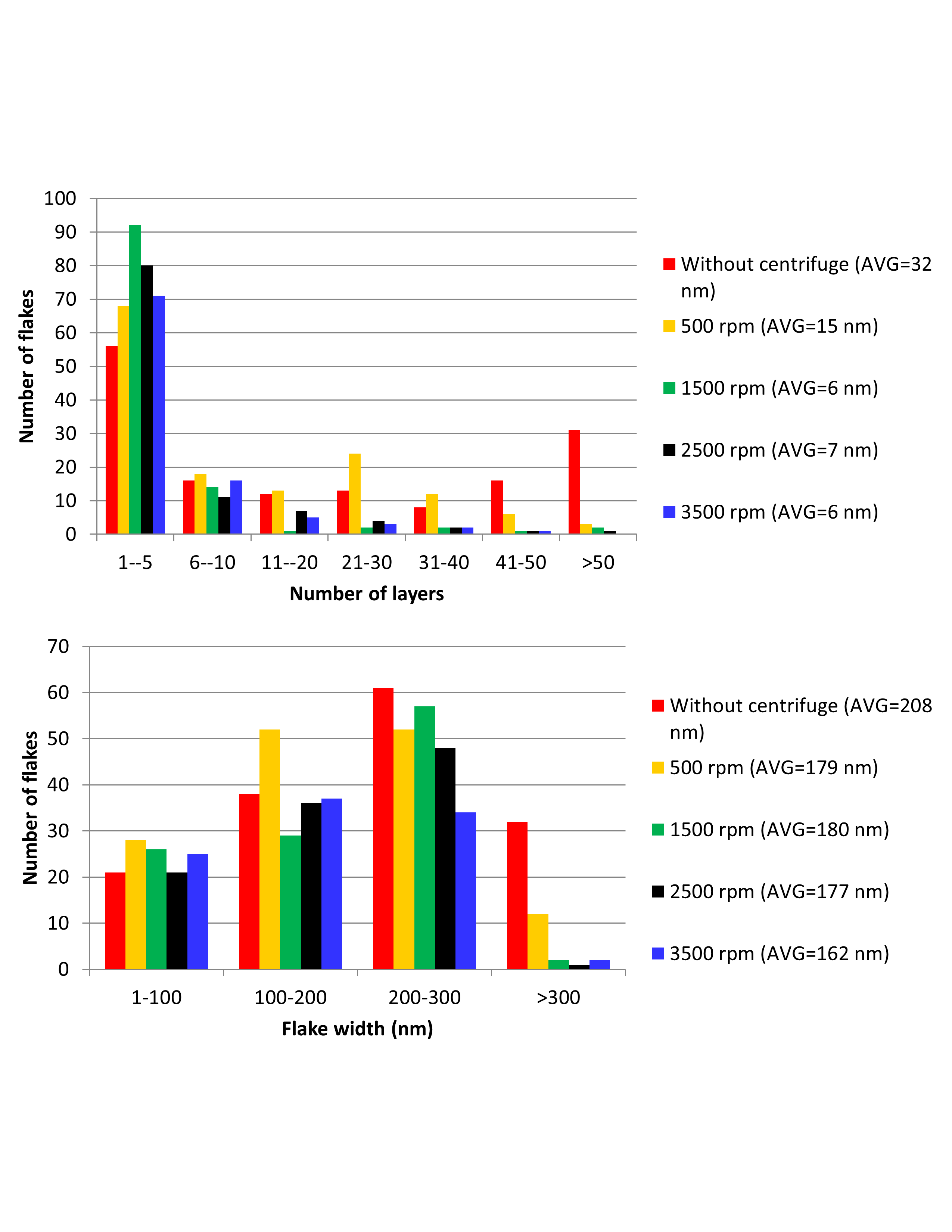


# **Figure S_3_.** AFM statistics on width and height of WS_2_ flakes for different centrifuge speeds.


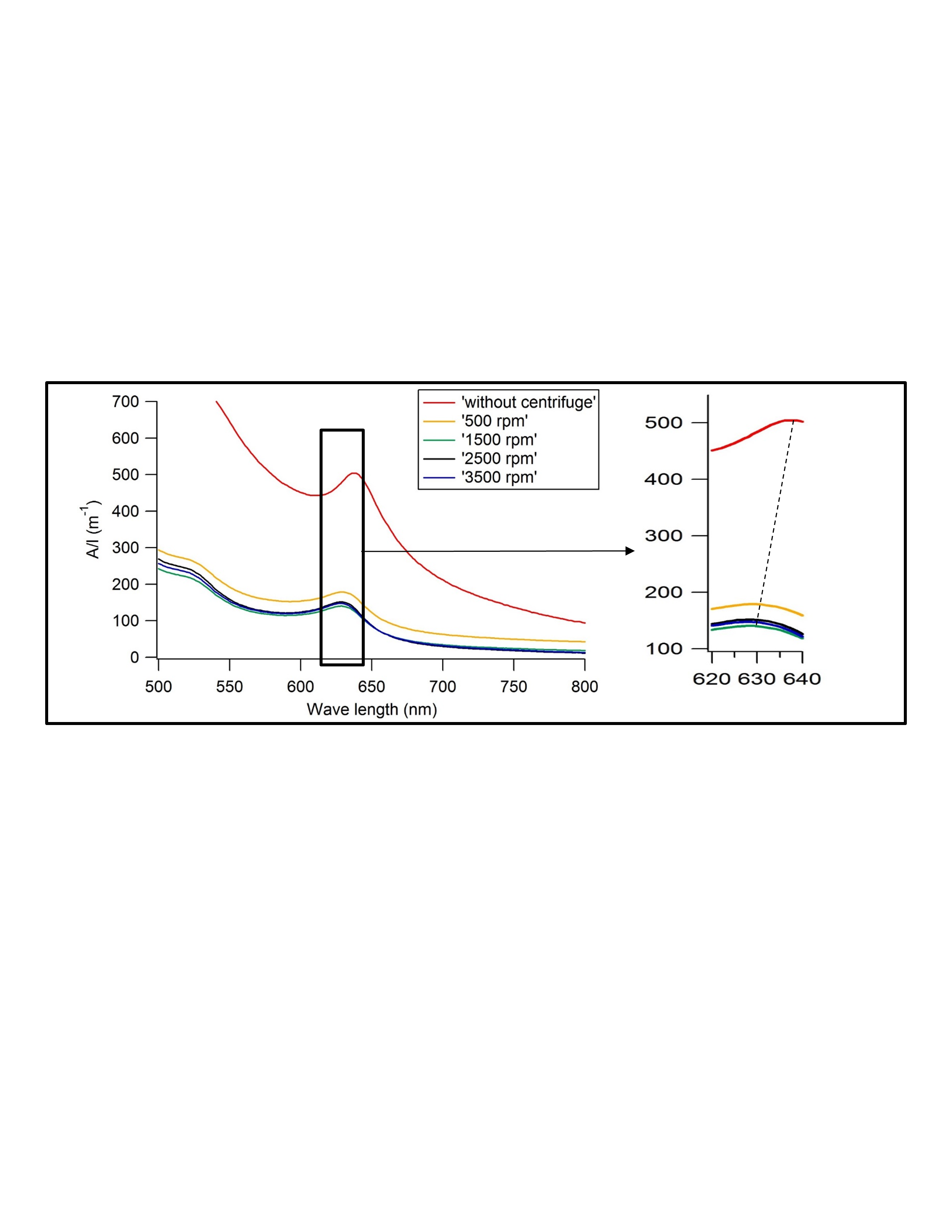


# **Figure S_4_.** Absorption of exfoliated WS_2_ solutions at DMF which are centrifuged with different speeds.


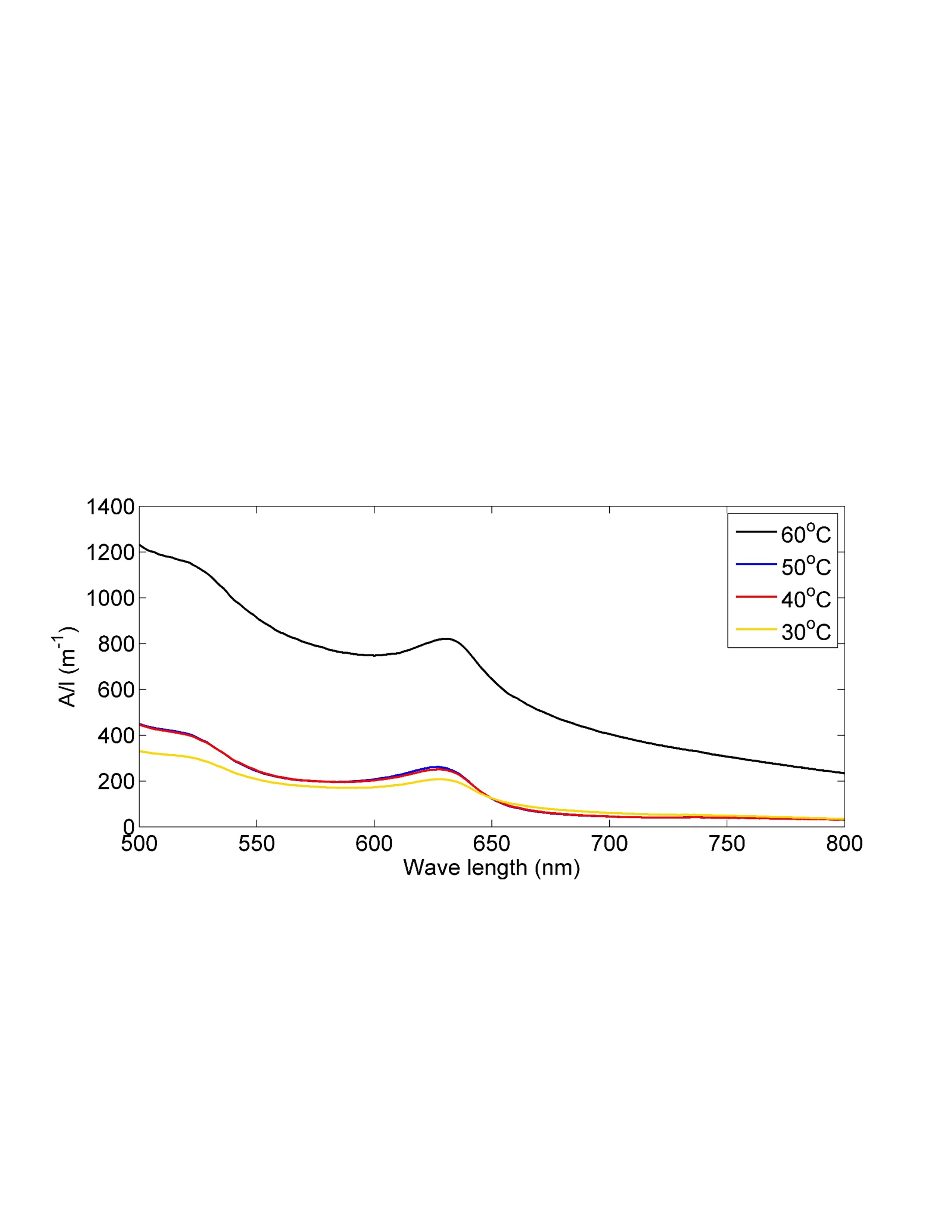


# **Figure S_5_.** Absorption of WS_2_ solutions at DMF exfoliated at different temperatures.


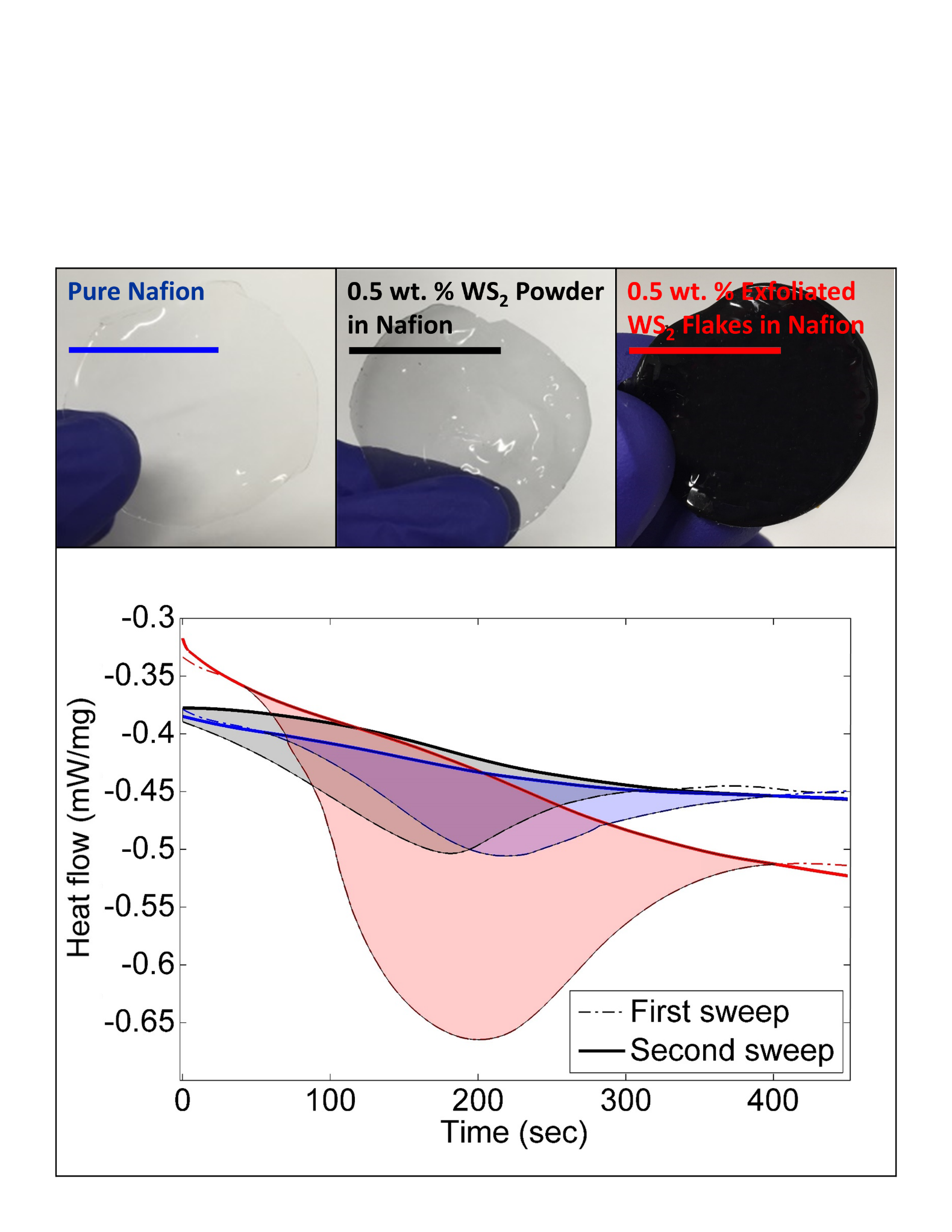


# **Figure S_6_.** Thermograph of WS_2_ –Nafion composites under ambient condition. This figure shows that the large peak around 80^o^C which shows the evaporation of the water inside the Nafion composite disappears in the second temperature sweep. The shaded area inside each curve represents the evaporation enthalpy of desorbed water.


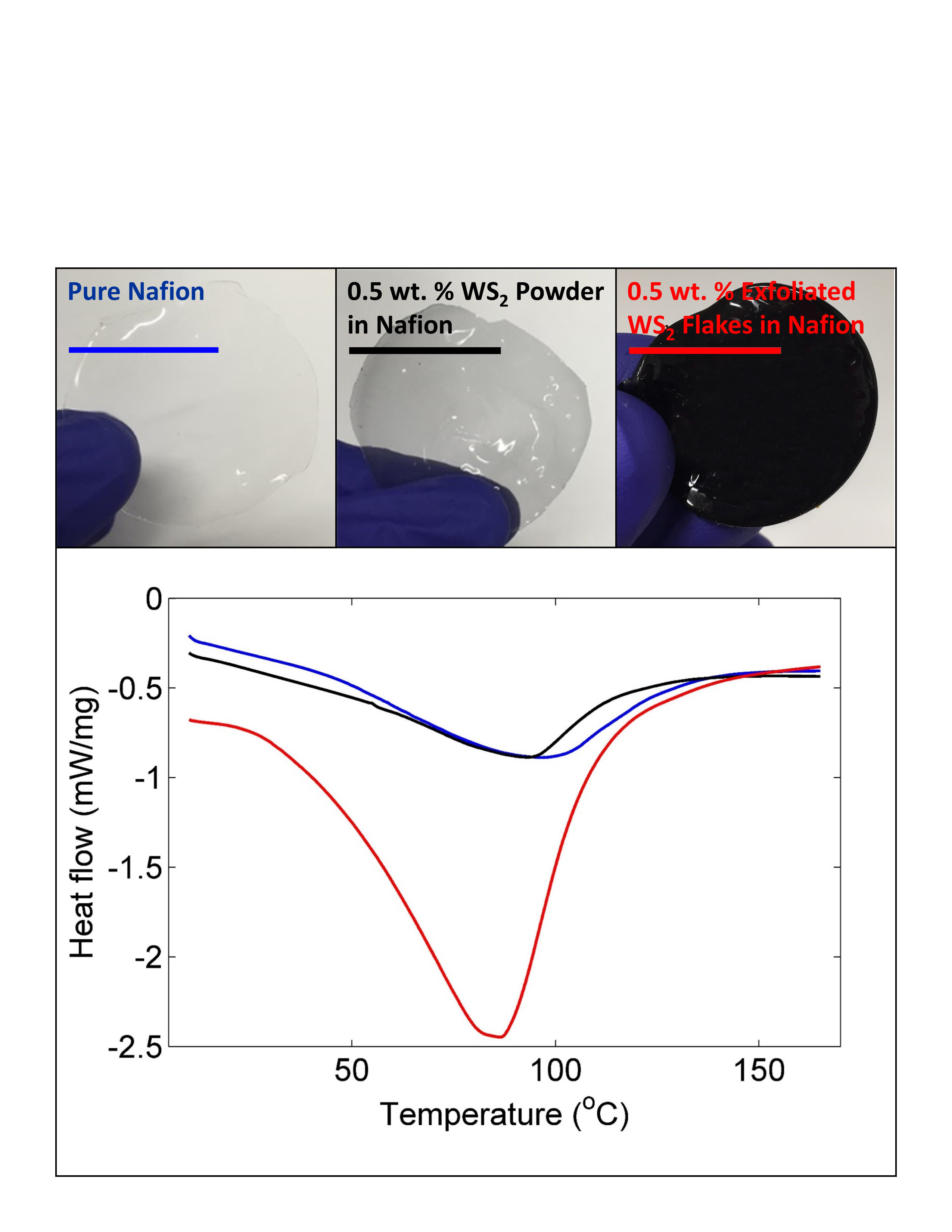


# **Figure S_7_.** Thermograph of the WS2- Nafion composites while they were fully wet.


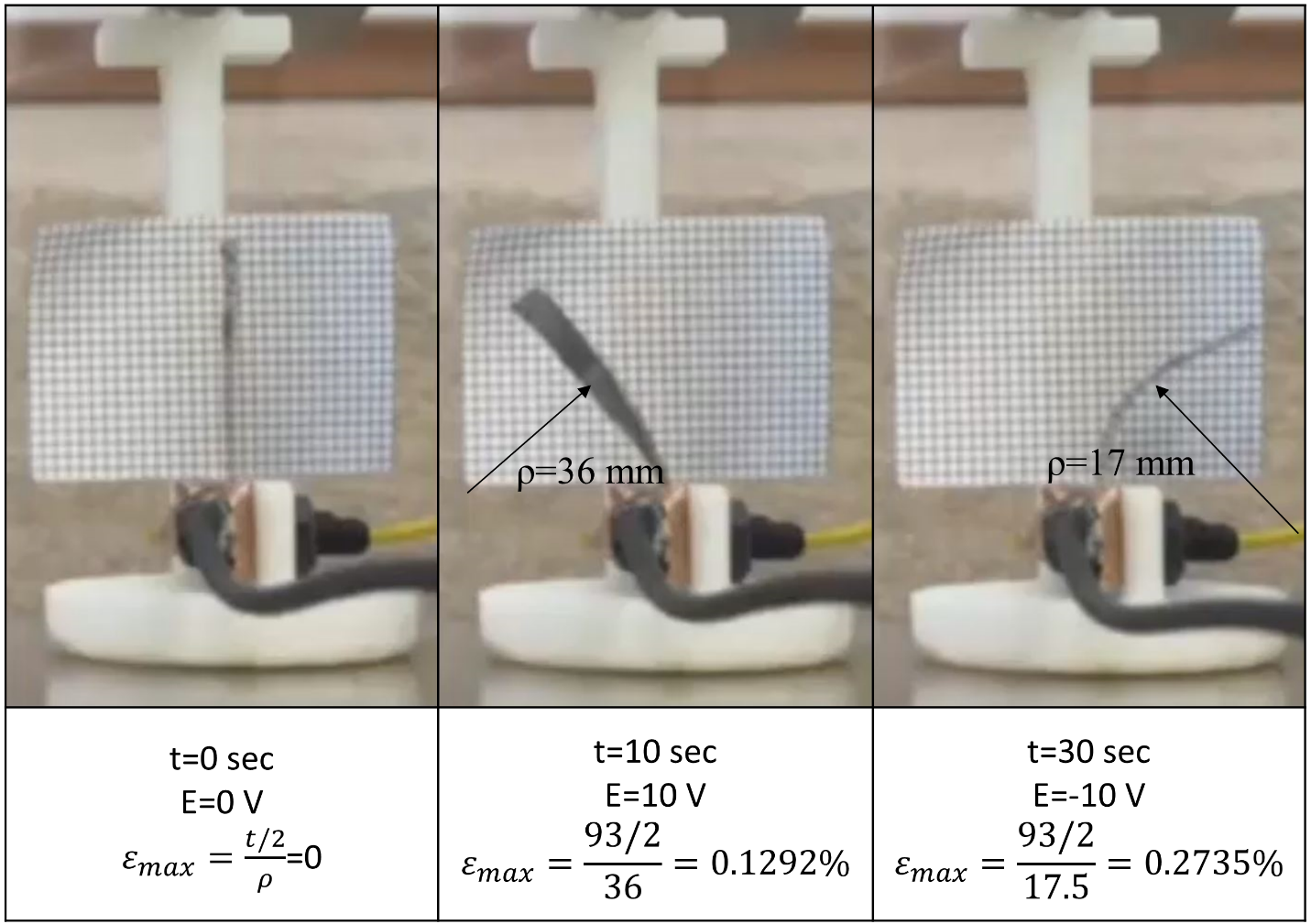


# **Figure S_8_.** 3 frames of a cyclic voltammetry test on the 0.5 wt. % exfoliated WS_2_ in Nafion composites while the scanning rate was 10 V/s and the maximum electric field was 10 volts. The length of the actuator is 20 mm and the maximum strain amplitude was 0.2735%.


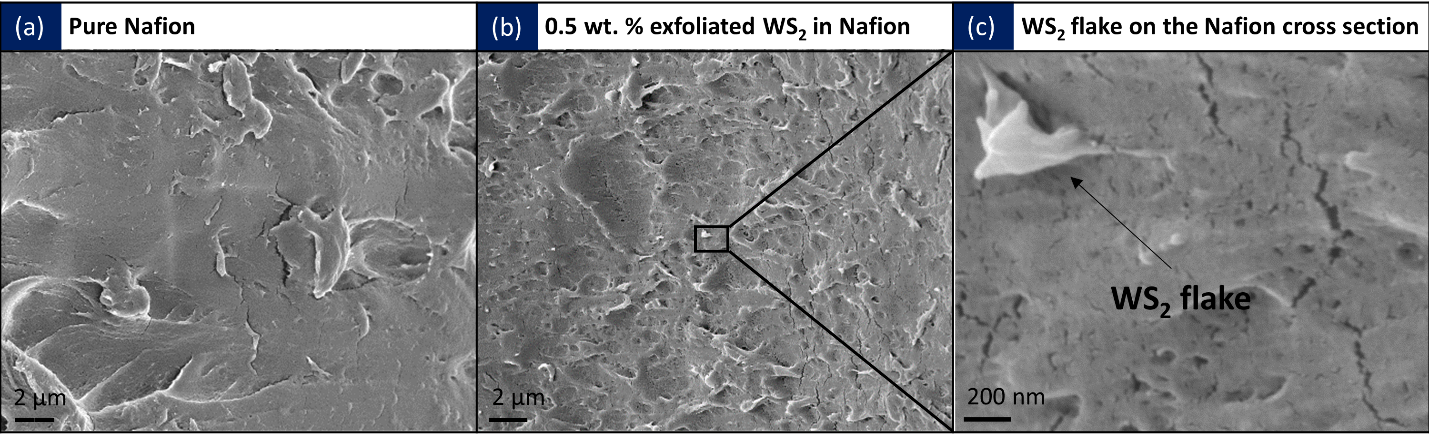


# **Figure S_9_.** SEM image of Nafion composites, (a) cross section of pure Nafion at 5K magnification, (b) cross section of 0.5 wt. % exfoliated WS_2_ in Nafion at 5K magnification, (c) cross section of 0.5 wt. % exfoliated WS_2_ in Nafion at 40K magnification showing the WS_2_ flake.
